# Translation and validation of Malay version of NIOSH worker well-being questionnaire (WellBQ)

**DOI:** 10.1371/journal.pone.0322451

**Published:** 2025-05-09

**Authors:** Nionella Stephen Sampil, Aziah Daud, Suhaily Mohd Hairon

**Affiliations:** Department of Community Medicine, School of Medical Sciences, Universiti Sains Malaysia, Kubang Kerian, Kota Bharu, Kelantan, Malaysia; STIKES Wira Medika PPNI Bali: Sekolah Tinggi Ilmu Kesehatan Wira Medika PPNI Bali, INDONESIA

## Abstract

The NIOSH Worker Well-Being Questionnaire (WellBQ) offers a comprehensive framework to evaluate worker well-being across five domains: work evaluation, workplace policies, physical environment and safety, health status, and home/community influences. In Malaysia, traditional occupational safety and health (OSH) initiatives have primarily focused on workplace hazards, often neglecting broader psychosocial and organizational factors. To address this gap, this study adapted and validated the Malay version of the WellBQ for healthcare workers, ensuring cultural and contextual relevance. A rigorous translation process, including forward and backward translation, expert panel reviews, and pilot testing, was conducted to retain the original framework while addressing local nuances. Psychometric evaluation involved 366 healthcare workers from Hospital Universiti Sains Malaysia, employing Confirmatory Factor Analysis (CFA) to assess model fit, internal consistency, and construct validity. The Malay WellBQ demonstrated robust psychometric properties, with a Content Validity Index (CVI) of 0.92 and a Face Validity Index (FVI) of 0.98, reflecting high relevance and clarity. CFA confirmed an acceptable model fit (RMSEA = 0.050, CFI = 0.887, TLI = 0.877) and strong internal consistency (CR > 0.7). Convergent validity was observed across most subdomains, although some Average Variance Extracted (AVE) scores fell below 0.5, highlighting areas for refinement. Discriminant validity was achieved within domains but revealed overlaps between some domains, suggesting interconnected constructs. The Malay WellBQ is a reliable and culturally relevant tool for assessing worker well-being, offering actionable insights for workplace policy and intervention development. Further refinements are recommended to enhance construct validity across domains.

## Introduction

Worker well-being encompasses a multifaceted spectrum of health, including physical, mental, emotional, and social dimensions, within the workplace context. In Malaysia, traditional approaches to occupational safety and health (OSH) have focused predominantly on managing specific workplace hazards, such as needlestick injuries, communicable diseases (e.g., Hepatitis B, Hepatitis C, HIV, and TB), and workplace violence against healthcare workers [1–5]. These initiatives are often fragmented, with responsibilities distributed among entities like the Department of Occupational Safety and Health and the Occupational Safety and Health Unit under the Ministry of Health [6,7]. This siloed approach limits the development of integrated strategies that holistically address worker well-being.

Programs like KOSPEN PLUS, later rebranded as KOSPEN WOW, have emphasized lifestyle interventions targeting nutrition, physical activity, smoking cessation, and mental health. However, these programs do not comprehensively address broader workplace well-being dimensions, such as organizational culture, psychosocial risks, and non-work factors. Such gaps underscore the urgent need for frameworks that integrate workplace policies, safety climates, and psychosocial determinants to enhance worker health and productivity. The evolving nature of work, influenced by rapid technological advancements, demographic shifts, and societal expectations [8,9], further emphasizes the need for holistic approaches to worker well-being.

Global frameworks like the Total Worker Health™ (TWH) initiative offer a blueprint for integrating organizational policies with individual behaviors to foster thriving and sustainable workforce environments [10]. This approach links well-being to organizational performance, aligning with the PERMA model’s pillars—Positive Emotion, Engagement, Relationships, Meaning, and Accomplishment [11]. By addressing both subjective experiences and objective conditions, these frameworks advocate for strategies that harmonize personal fulfillment with organizational goals.

Building on the TWH program, Chari et al. (2018) propose a conceptual framework that integrates both work and non-work contexts, defining worker well-being as a dynamic interplay of subjective perceptions (e.g., job satisfaction and emotional balance) and objective determinants (e.g., workplace policies and access to resources)[12,13]. This framework emphasizes the interconnectedness of work and personal life, highlighting the role of home, community, and societal influences in shaping well-being. As work-life boundaries continue to blur, fostering comprehensive well-being requires shared responsibilities among organizations, communities, and individuals [12,14].

The NIOSH Worker Well-Being Questionnaire (WellBQ) exemplifies this holistic approach, offering a multidimensional assessment tool that evaluates workplace and broader determinants of well-being [15]. The NIOSH Worker Well-Being Questionnaire (WellBQ) stands out as a comprehensive multidimensional tool that assesses physical, emotional, and social health across both work and non-work contexts. Unlike tools like COPSOQ II, which focus on psychosocial risks [16], or the Abundance Index for Workers (AIW) [17], which emphasizes societal resources, the WellBQ integrates both subjective (e.g., emotional well-being) and objective (e.g., workplace safety) metrics. Tools like PRISMA (Psychosocial Risk Assessment & Management at the Workplace) [18] target psychosocial risk management by addressing job demands and organizational culture. In contrast, the NIOSH WellBQ provides a broader framework that evaluates multidimensional well-being, including non-work influences, offering actionable insights into policies, safety climates, and interventions due to its robust psychometric properties[15]. Adaptations of the WellBQ for cultural relevance, such as translations further enhance its applicability across diverse settings, providing insights that inform inclusive policies and interventions.

In contrast, programs like KOSPEN WOW excel in promoting healthy lifestyle behaviours through workplace-based interventions but lack the comprehensive scope of tools like the WellBQ which evaluates psychosocial risks, workplace policies, and safety climates, making it a more holistic tool for understanding worker well-being [19]. Aligning initiatives like KOSPEN WOW with global frameworks, such as TWH and WHO guidelines, could amplify their impact by addressing psychosocial and environmental determinants of well-being. This integration would strengthen workforce resilience, improve organizational performance, and contribute to sustainable health outcomes. Furthermore, the NIOSH WellBQ reflects the shift in OSH from a traditional biomedical focus to a biopsychosocial model, as advocated by Schulte et al. (2022) [20]. This multidimensional approach aligns with broader frameworks, such as the World Health Organization’s (WHO) definition of well-being, which highlights the role of job satisfaction, mental health, workplace safety, and work-life balance as contributors to productivity and quality of life [21].

In Malaysia, factory workers generally report high job satisfaction, indicating a positive work environment [22]. However, work-family conflicts negatively affect female academicians’ well-being, highlighting the need for management support [23]. Additionally, Workplace sexual harassment remains a serious issue, causing emotional distress and increasing suicide risk, especially among vulnerable women [24,25]. The health of workers, particularly healthcare workers, is crucial. A multinational study found high levels of burnout, anxiety, and depression among healthcare workers, especially doctors, nurses, and clinical staff [26]. The COVID-19 pandemic further exposed significant health impacts on Malaysian healthcare workers, including high rates of suicidal ideation and depression, underscoring the need for better well-being measures and interventions [27]. Workplace conditions, such as job demands, stress, and organizational changes, significantly impact well-being, especially in high-risk sectors like construction and healthcare settings. Psychosocial and environmental factors, including workload, autonomy, and support, are key to worker health and align with the NIOSH WellBQ’s holistic framework. Enhancing occupational health also supports the UN’s Sustainable Development Goal 3 on good health and well-being [28].

By embracing comprehensive well-being frameworks that integrate work and non-work domains, organizations can create environments that support health, engagement, and productivity in an evolving work landscape. This study seeks to validate and assess the reliability of the Malay version of the NIOSH WellBQ. The research evaluates the consistency and construct validity of the adapted instrument among healthcare workers in Kelantan hospital, Malaysia. Tools like the NIOSH WellBQ provide actionable insights for improving worker health and organizational outcomes, reinforcing the importance of holistic strategies in addressing the complexities of modern work-life dynamics.

## Methods

### Study design and population

The study utilized a cross-sectional design to validate the Malay version of the NIOSH Worker Well-Being Questionnaire (WellBQ). Conducted at Hospital Universiti Sains Malaysia (HUSM) in Kelantan, Malaysia, the study took place from November 2022 to June 2023. A total of 366 healthcare workers, including doctors, nurses, medical assistants, and other professionals, were randomly sampled for the validation process. Participants were required to be proficient in Malay and have at least six months of work experience.

Data collection occurred between February and April 2023 using a web-based survey. The questionnaire was distributed via Google Forms through email and WhatsApp to the selected participants. Both online and manual face-to-face methods were employed to ensure accessibility and maximize participation.

Participants provided informed consent electronically through Google Forms before completing the survey. The consent process included a detailed explanation of the study objectives, procedures, confidentiality measures, and the voluntary nature of participation. Participants indicated their agreement by selecting a checkbox before accessing the questionnaire. The use of Google Forms proved efficient, ensuring a high response rate and timely validation [29].

The collected responses were securely stored in a password-protected file and analyzed using Microsoft Excel, and exported for analysis in SPSS version 27 and SPSS AMOS. Confirmatory Factor Analysis (CFA) was performed to validate the questionnaire, ensuring the reliability and structure of the Malay version of the NIOSH WellBQ.

**Sample size.** The sample size was calculated using a web-based calculator [30] by Arifin WN, based on the Structural Equation Modelling – Comparative Fit Index (CFI) method. After adjusting for a 10% non-response rate, the required sample size was 366 respondents. Comrey and Lee’s standards for CFA model adequacy, which categorize sample sizes of 100 as poor, 200 as fair, 300 as good, 500 as very good, and 1000 as more as excellent [31]. Hence, a sample size is considered adequate for this study.

### Questionnaire

The NIOSH WellBQ, consisting of 126 items, was translated into Malay to enhance its applicability for local use. The questionnaire includes 16 scales, five indices, and 31 single items, organized into five key domains: (1) Work Evaluation and Experience refer to how individuals perceive their work life, including job satisfaction, meaningfulness, engagement, and emotional well-being at work; (2) Workplace Policies and Culture refers to organizational policies, programs and practices that impact worker well-being; (3) Workplace Physical Environment and Safety Climate refers to the physical and safety aspects of the workplace, including both physical and psychological safety; (4) Health Status refer to individual’s physical and mental health and overall health functioning; and (5) experiences activities related to Home, community, and society refer to non-work aspects of life, like home and community factors, that influence well-being. Its robust psychometric properties, such as strong internal consistency (Cronbach’s alpha > 0.8) and good model fit (CFI and TLI > 0.93), ensure reliability and validity in occupational health research. Concurrent, convergent, and discriminant analyses further support its construct validity [15]. The tools, originally developed for use in United States (full questionnaire available at the CDC NIOSH website via: https://www.cdc.gov/niosh/docs/2021-110/default.html).

### Translation process and psychometric analysis

#### Translation process.

The NIOSH Worker’s Well-Being Questionnaire (WellBQ) was translated into Malay by employing a back-to-back methodology to ensure cultural relevance and linguistic accuracy following guidelines from [32,33]. Two bilingual translators—one with an education background and the other a public health professional—performed the forward translation to ensure cultural relevance. An expert panel then reviewed it for conceptual, semantic, operational, and measurement equivalence. A backward translation was conducted by another pair of bilingual translators to identify any discrepancies. The process involved multiple reviews and comparisons with the original English version by expert panels (Table 1), resulting in a harmonized final version. This thorough translation process ensures the translated questionnaire effectively measures worker well-being in Malaysia, maintaining the integrity of the original.

**Table 1:** Credentials of Expert Panels.

Role	Credentials
Expert panel 1 Expert panel 2	Bilingual (Bahasa Malaysia and English) medical officer with experience in English-speaking culture and public health professional Bilingual (Bahasa Malaysia and English) medical officer with experience in English-speaking culture and public health professional
Expert panel 3	Bilingual Master of Business graduate with experience working in English-speaking culture
Expert panel 4	Bilingual public secondary schools that teach English courses and have experiences in English-speaking culture

#### Content validation.

The pre-final Malay version of NIOSH WellBQ underwent content validation to ensure its item effectively represented in the intended constructs. A panel of four experts in public health, occupational health and health system management reviewed the questionnaire using a virtual content validation form through google form. Each expert rated the relevance of 126 items into each domains after written consent obtained, with the Item-Level Content Validity (I-CVI) and Scale-Level Content Validity Index (S-CVI) calculated to assess content validity. The I-CVI considered acceptable at 0.78 or higher, S-CVI/Ave at 0.9 or higher, and S-CVI/UA at 0.8 or higher for strong content validity [34]. Satisfactory content validity index indicate proceeding with face validity.

#### Face validation.

Face validation for the prefinal Malay version of NIOSH WellBQ involved assessing the clarity and comprehension of the questionnaire items. A panel of 30 healthcare workers from Hospital Universiti Sains Malaysia (HUSM) reviewed the tool using a response process validation form. Scores were recoded, and an average score (S-FVI/Ave) and high-rating proportion (S-FVI/UA) were calculated, with values above 0.8 indicating satisfactory face validity [35]. Feedback was collected through group meetings and independent scoring, with all comments considered for refining the questionnaire.

#### Psychometric analysis.

The validation process for the Malay version of the NIOSH WellBQ involved multiple steps to ensure the questionnaire’s reliability and validity. First, individual items were analyzed to assess response pattern, score ranges, and the presence of floor or ceiling effects. Items that are indices or binary response items were not subjected for Confirmatory Factor Analysis such as subdomain construct availability of health programs at work, availability of job benefits, work-related sexual harassment, work-related physical violence. CFA was conducted on 13 factors with 82 items excluding 44 items that were binary responses. Assumptions for CFA were thoroughly checked, including univariate and multivariate normality, multicollinearity, and model fit. Univariate normality was evaluated through skewness and kurtosis, with severe non-normality flagged at skewness >2.0 or kurtosis > 7.0. Multivariate normality was assessed using Mardia’s test through online tool Webpower at http://webpower.psychstat.org/models/kurtosis/ [36]. Outliers were identified using Mahalanobis distance in SPSS AMOS.

Validity and reliability are essential for ensuring the accuracy and consistency of a measurement model. Validity encompasses convergent validity, construct validity, and discriminant validity. Construct validity of Malay NIOSH WellBQ via model fit indices was evaluated using indices such as Root Mean Square of Error Approximation (RMSEA<0.08), Goodness of Fit Index (GFI > 0.90), and Comparative Fit Index (CFI > 0.90), ensuring satisfactory absolute, incremental and parsimonious fit. Convergent validity is observed when Composite reliability (CR) is higher than the Average Variance Extracted (AVE) score of 0.5 or higher for each factor or subdomain (e.g satisfaction,support at work). Discriminant validity ensures independence between five domains of NIOSH WellBQ with inter-factor correlations below 0.85 which can be obtained through square root of AVE [37]. Reliability was assessed through Composite Reliability (CR) and Average Variance Extracted (AVE) for each subdomain, measures internal consistency, with acceptable thresholds of ≥0.7 for CR and 0.5 for AVE [38]. Composite reliability (CR ≥ 0.70) offers a more robust measure than Cronbach’s Alpha [39,40]. CR values ≥ 0.70 indicate acceptable reliability, ≥0.80 suggest good reliability, and ≥0.90 imply excellent reliability, although values exceeding 0.90 may indicate overfitting [41]. CR is preferred over Cronbach’s Alpha in Structural Equation Modeling (SEM) due its robust and comprehensive analytical capabilities [42]. In this study, both CR and AVE was calculated using Microsoft Excel by including number of items and factor loadings of all items in the model, following a specific formula. These analyses, conducted using IBM SPSS Statistics version 27 and IBM SPSS Amos version 29. Table 2 summarizes the fit indices and their cut off value [39,41].

**Table 2:** Three categories of model fit and their cut-off value.

Name of category	Name of Index	Cut Off Value
**Absolute Fit**	Chi-Square	P-value >0.05 (Not applicable for large sample)
	RMSEA^1^	<0.08
	GFI^2^	>0.90
**Incremental fit**	AGFI^3^	>0.90
	CFI^4^	>0.90
	TLI^5^	>0.90
**Parsimonious fit**	Chisq/df^7^	<3.0

1 - Root Mean Square of Error Approximation

2 – Goodness of Fit Index

3 – Adjusted Goodness of Fit Index

4 – Comparative Fit Index

5 – Tucker Lewis Index

**Measures.** The demographic questionnaire (Part A) collected data on age, sex, ethnicity, education, occupation, and job duration. The WellBQ (Part B) includes five domains: work evaluation and experience, workplace policies and culture, physical environment and safety, health status, and home/community as shown in **Table 3** [15]. The questionnaire utilizes a combination of 4-point and 7-point Likert scales across various domains. It includes both agreement-based scales (ranging from 1 - Strongly Disagree to 4 - Strongly Agree) and frequency-based scales (ranging from 1 - Never, 2 - Almost Never, 3 - Rarely, 4 - Sometimes, 5 - Often, 6 - Very Often, to 7 - Always). Although the WellBQ lacks norms for comparing worker well-being across populations and does not provide summary score algorithms, it still offers valuable insights by analyzing individual responses and scale scores.

**Table 3:** Items and Subscales in NIOSH Worker’s Well-being Questionnaire (WellBQ).

Domain	Subdomain	Items	Subdomain constructs
**Work evaluation and experience (16 items)**	Satisfaction	1	Job Satisfaction
		1	Wage Satisfaction
		1	Benefits Satisfaction
		1	Advancement satisfaction
	Support at work	1	Supervisor Support
		1	Coworker Support
	Evaluation of work conditions	1	Job security
		1	Job autonomy
		1	Time paucity/work overload
	Meaning	2-item scale	Meaningful work
	Affect	4-item scale	Work-related Positive Affect
		4-item scale	Work-related Negative Affect
	Fatigue	1	Work related fatigue
	Job Engagement	3-item scale	Job engagement
**Workplace Policies and Culture** **(14 items)**	Supportive work culture	5-item scale	Supportive Work Culture
		1	Management trust
	Health Culture at Work	2-item scale	Health Culture at Work
		7-item index	Availability of health programs at work*
	Benefits	14-item index	Availability of job benefits*
	Organization of work and life	1	Work and non-work conflict
		1	Nonwork to work conflict
		2-item scale	Workplace/schedule flexibility
**Workplace physical environment and safety climate** **(10 items)**	Safety Climate	1	Overall workplace safety
		6-item scale	Workplace safety climate
	Physical work environment satisfaction	4-item scale	Physical work environment satisfaction
	Interpersonal conflict and incivility	3-item scale	Discrimination
		1	Work-related sexual harassment*
		1	Work-related physical violence*
		2-item scale	Work-related bullying*
**Health Status** **(23 items)**	General Health	1	Overall health
	Physical Health	1	Days of poor physical health*
		9-item index	Chronic health conditions*
		1	Insomnia*
	Mental Health	1	Days of poor mental health*
		4-item scale	Overall stress
		4-item scale	Poor mental health (Depression)
	Health behaviour	2-item scale	Physical Activity*
		5-item index	Tobacco use*
		1	Alcohol consumption*
		1	Risky drinking*
		1	Healthy diet*
		1	Sleep hours*
		1	Sleepy at work
	Functioning	1	Cognitive functioning limitations
		1	Work limitations
		4-item scale	Productivity
	Injury	1	Work-related injury*
		1	Injury Consequences*
**Home, community, and society (5 items)**	Life satisfaction	1	Life satisfaction
	Financial insecurity	2-item scale	Financial insecurity
	Social relationships	1	Support outside work
	Activities outside of work	7-item index	Activities outside of work

* Items that were not subjected to CFA

### Ethical considerations

The research was approved by the Human Research Ethics Committee of Universiti Sains Malaysia (USM/JEPeM/22110724), ensuring ethical standards were met. Participants provided informed consent, understanding the study’s purpose, procedures, and any potential risks or benefits. Confidentiality was protected, encouraging honest responses, and participation was voluntary without coercion. Data access was limited to the authors and supervisors, and reporting was conducted anonymously, without requiring personal identification.

## Results

### Demographic Characteristics of Respondents

A total 370 participants took part, response rate was 98.9% (366 participants), and 1.1% (4 participants) did not respond to the questionnaire. Table 4 summarizes the socio-demographic characteristics of the participants involved in Confirmatory Factor Analysis (CFA).

**Table 4.** Characteristics of respondents (n = 366).

Variables		n (%)
Age (Years)		
	18–29	54 (14.8)
	30–44	260 (71.0)
	45–60	52 (14.2)
Sex		
	Male	88(24.0)
	Female	278 (76.0)
Ethnicity		
	Malay	357 (97.5)
	Chinese	6 (1.6)
	Indian	3 (0.8)
	Other Bumiputeras	0(0)
Education level		
	Primary School	0 (0)
	Secondary School	38 (10.4)
	Diploma	234 (63.9)
	Bachelor’s Degree or higher	94 (25.7)
Occupation		
	Specialist	7 (1.9)
	Medical Officer	15 (4.1)
	House Officer	3 (0.8)
	Dental Officer	16 (4.4)
	Dental nurse	6 (1.6)
	Pharmacist	15 (4.1)
	Assistant Pharmacy	24 (6.6)
	Medical Assistant	36 (9.8)
	Lab Science officer	2 (0.5)
	Lab Technician	11 (3.0)
	Nurse	193 (52.7)
	Attendants	38 (10.4)
Type of Occupation		
	Permanent post	334 (91.3)
	Contract post	32 (8.7)
Job Duration (Years)		
	Less than one year	20 (5.5)
	1–10 years	139 (38.0)
	11–20 years	142 (38.8)
	More than 20 years	65 (17.8)

### Psychometric validation of the worker’s WellBQ

The Content Validity Index (CVI) achieved satisfactory levels with a Scale-Level CVI/Average (S-CVI/Ave) of 0.92, meeting the required standards. Based on expert feedback, revisions were made to enhance the accuracy of the translation. In the face validation phase, 30 experienced medical officers served as raters, and the Face Validity Index (FVI) was calculated at 0.98, indicating high relevance, clarity, and comprehensibility of the questionnaire items. Univariate normality was confirmed in SPSS, with skewness peaking at 2.36. However, multivariate normality showed significant non-normality with a kurtosis value of 47.8. Outliers were identified using Mahalanobis distance in SPSS AMOS, marking 22 observations as outliers. Due to the substantial deviation from normality, the Maximum Likelihood Robust Estimator (MLR) was used. Bootstrapping, a statistical technique that involves resampling from the existing dataset with replacement was also employed to enhance analysis robustness by generating a new sampling distribution. The CFA results were validated by comparing them with outcomes from the bootstrapped data. [43]

To determine the best-fitting model, three models were evaluated using Confirmatory Factor Analysis (CFA) on 82 likert scales. EFA is generally recommended before CFA in new instrument validation to assess latent structure [44]. However, we proceeded directly with CFA based on strong theoretical underpinnings and prior validation of the original instrument [39]. The initial model showed poor fit indices, with standardized factor loadings ranging from 0.032 to 0.974 and some negative loadings, indicating inverse relationships. This was attributed to under-factoring, leading to a revised model that incorporated both 1st and 2nd order factors. Upon revising the model, initial issues of over-factoring at the first-order level were addressed by consulting experts and reducing the number of factors in various domains: from six to four factors in the Work Evaluation and Experience domain (specifically items under subdomain support at work, evaluation of work conditions and meaning consolidated under satisfaction subdomain), three to two factors in the Workplace Policies and Culture domain (specifically items under subdomain health culture at work and benefits consolidated under supportive work and health culture), and consolidating the Home, Community, and Society domain into a first order factor. These adjustments led to an improved fit in the second model, with factor loadings now ranging from 0.017 to 0.979. However, despite improvements, fit indices still did not meet the acceptable thresholds.

Ten items in the second model exhibited low factor loadings, ranging from 0.017 to 0.240 [45]. After consulting with experts, it was determined that these modest loadings might cause cross-loadings with other factors. Further discussions and expert consultations, led to the reassignment of Item 8 (Job Autonomy) to another domain—Domain 2 (Workplace Policies and Culture) under F2 (Supportive Work Culture) to enhance domain consistency. Additionally, nine items—Item S9 (Time paucity/work overload), S29 (Workplace/schedule flexibility), S41 (Overall health), S58 (Sleepy at work), S59 (Cognitive functioning limitations), S64 (Life satisfaction), S65 (Financial insecurity), S66 (Financial insecurity), and S67 (Support outside of work)—were iteratively removed due to their very low factor loading estimates, all of which were below 0.3 [44].

While assessing for convergent and discriminant validity, eight items having lower factor loadings of 0.5 were advised for further removal in order to improve model fit [38,39]. The 8 items such as S2 (wage satisfaction), S3 (benefits satisfaction), S4 (advancement satisfaction), S12F (Work-related negative affect (angry), S13 (Work-related fatigue), S30 (Workplace flexibility), S31 (overall workplace safety) and S33D (physical work environment satisfaction). This lead to further improvement in final model fit. The model was not further constrained by correlating errors between two items with high modification indices due to insufficient theoretical evidence and potential complications in the study related to the types of analytical methods employed [44,46]. Eventually, the validated Malay NIOSH WellBQ was composed of 109 items (65 Likert scales and 44 items of categorical response).

Table 5 shows a summary of the fit indices for suggested models. As regard to the fit indices, initial model and second model did not achieve the standard values, with poor factor loadings for the 17 aforementioned items, as described above. The final model successfully achieved an acceptable fit for Worker’s WellBQ indicated by the CFA (RMSEA = 0.050, GFI = 0.736, CFI = 0.887, TLI = 0.877, χ ^2^/ df = 1.895). Other fit indices like CFI, TLI, and GFI indicated a relatively weak model fit. Nonetheless, when considering all indices together, the model demonstrated an acceptable level of fit despite its complex model.

**Table 5.** Model fit Indices of the Worker’s WellBQ.

**Absolute fit**	**RMSEA**	0.084	0.054	0.050
	**GFI**	0.481	0.697	0.785
**Incremental fit**	**AGFI**	0.453	0.678	0.759
	**CFI**	0.540	0.795	0.887
	**TLI**	0.527	0.788	0.877
**Parsimonious fit**	**χ** ^**2**^**/ df**	3.583	2.060	1.895

DOF: degree of freedom; RMSEA: root mean square error of approximation; CFI: comparative fit index; TLI: Tucker-Lewis index; GFI: Goodness of fit index

After finalizing the measurement model, Composite Reliability (CR) and Average Variance Extracted (AVE) was calculated and the results are displayed in Table 6. The CR values exceeded the 0.7 threshold [38,46], affirming the reliability and internal consistency of the latent constructs, thus confirming the final model’s capacity to measure the intended construct accurately.

**Table 6:** Results of Composite Reliability (CR) and Average Variance Extracted (AVE) for Final Model.

Domain (2^nd^ order Factor)	Subdomain (1^st^ order Factor)	Composite Reliability (CR)	Average Variance Extracted (AVE)
Work evaluation and experience (D1)	Satisfaction	0.747	0.334
	Work-related Positive Affect	0.963	0.867
	Work-related Negative Affect)	0.735	0.515
	Job Engagement	0.755	0.510
Workplace Policies and Culture (D2)	Supportive work and health culture	0.918	0.561
	Organization of work & life	0.753	0.604
Workplace Physical environment and safety climate (D3)	Workplace Safety Climate	0.924	0.671
	Physical Work Environment satisfaction	0.802	0.575
	Interpersonal conflict and incivility	0.781	0.550
Health Status (D4)	Overall stress	0.857	0.601
	Poor Mental Health	0.889	0.668
	Work Productivity	0.862	0.561
	Home, community, and society (D5)	0.851	0.454

Discriminant validity is supported by the discriminant validity index summary shown in Table 7, one can conclude that the discriminant validity for all constructs is achieved [44]. However, the discriminant validity between domains shown in Table 8 shows some issues of distinctiveness.

**Table 7:** Square root of AVE and inter-factor correlation as evidence of discriminant validity.

	Satisfaction	Work-related positive Affect	Work-related Negative Affect	Job Engagement	Supportive work & health culture	Organization of work & life	Workplace Safety Climate	Physical work environment satisfaction	Interpersonal conflict & incivility	Overall stress	Poor mental health	Work productivity	Home, community & society
Satisfaction	**0.555**												
Work-related Positive Affect	0.040	**0.931**											
Work-related Negative Affect	0.038	-0.055	**0.717**										
Job Engagement	-0.081	0.532	0.082	**0.714**									
Supportive work & health culture	0.041	0.225	0.092	0.462	**0.749**								
Organization of work and life	0.477	0.059	0.23	0.017	0.332	**0.777**							
Workplace safety climate	-0.003	0.245	0.123	0.279	0.441	0.204	**0.819**						
Physical Work Environment Satisfaction	0.036	0.293	0.134	0.449	0.567	0.308	0.523	**0.758**					
Interpersonal Conflict & Incivility	0.381	0.05	0.121	-0.048	0.026	0.421	0.023	0.053	**0.742**				
Overall Stress	-0.085	0.189	0.15	0.313	0.197	0.082	0.095	0.289	-0.123	**0.775**			
Poor Mental Health	0.116	0.263	0.129	0.522	0.336	0.101	0.158	0.313	0.04	0.554	**0.817**		
Work productivity	-0.168	0.127	0.096	0.358	0.104	0.011	0.038	0.211	-0.18	0.624	0.454	**0.749**	
Home, community & society	0.078	0.321	0.091	0.366	0.184	0.109	0.253	0.214	0.055	0.022	0.19	0.011	**0.674**

**Table 8:** Discriminant validity index summary (between domains).

	Workplace Evaluation & experience	Workplace policies and culture	Workplace physical environment and safety climate	Health status	Home, community and society
**Workplace Evaluation & experience**	**0.543**				
**Workplace policies and culture**	0.535	**0.67**			
**Workplace physical environment and safety climate**	0.575	0.797	**0.364**		
**Health Status**	0.551	0.326	0.364	**0.551**	
**Home, community & society**	0.434	0.217	0.302	0.083	**0.454**

## Discussion

### Final instrument of Malay version WelBQ

The final validated Malay NIOSH WellBQ consists of 109 items (65 Likert scales and 44 categorical items), retaining its multidimensional structure while adapting to Malaysia’s cultural and occupational context. Rigorous processes, including back-to-back translations, expert reviews, and pilot testing, ensured linguistic equivalence and content validity[47,48]. This methodical approach allowed the Malay WellBQ to retain the theoretical framework and constructs of the original NIOSH WellBQ while addressing culturally specific nuances relevant to Malaysian workers.

The psychometric evaluation of the Malay version of NIOSH WellBQ demonstrated strong reliability and validity, affirming its robustness as a multidimensional tool for assessing worker well-being. Composite reliability (CR) confirmed strong internal consistency, with all values exceeding 0.7 (Bagozzi and Yi, 1988). High CR values indicate the questionnaire’s stability in capturing constructs consistently across different contexts. Convergent validity achieved across most of subdomains, however, some Average Variance Extracted (AVE) values were below 0.5 threshold such as subdomain satisfaction and home, community & society. The Fornell & Larcker Criterion provided compensatory evidence of convergent validity through high CR values [49]. Low AVE values can result from construct overlap, multicollinearity, or item redundancy [50]. For future research, several recommendations to address low AVE, researchers should prioritize content validity by refining item wording and seeking expert judgment before modifying or eliminating items. Increasing the sample size can enhance statistical power, reducing standard errors and improving AVE estimates, leading to more stable factor loadings. When items exhibit high correlations across constructs, exploring a higher-order factor model can help better capture variance. Additionally, items contributing to multicollinearity and construct overlap should be either removed or reassigned if theoretically justified. Rather than relying solely on the Fornell-Larcker criterion, employing the HTMT ratio provides a more robust assessment of whether constructs are conceptually distinct [51]. Discriminant validity was also assessed, ensuring the construct were distinct and not overly correlated, which further enhance construct validity compared to other validated WellBQ [48]. This balance ensures that the Malay WellBQ remains a reliable and actionable tool for workplace assessments, enabling targeted interventions to improve organizational and worker outcomes.

The model fit indices for the Worker’s WellBQ showed significant improvement from the initial to final model, aligning with Hu and Bentler’s (1999) recommendation for using multiple indices in model assessment. The RMSEA improved from 0.084 to 0.050, indicating a shift from mediocre to close fit. The χ²/df ratio decreased from 3.583 to 1.895, reflecting better model balance. Incremental fit indices, CFI (0.540 → 0.887) and TLI (0.527 → 0.877), showed substantial progress, nearing the optimal range of 0.90–0.95 but still requiring refinement. The GFI increased from 0.481 to 0.785, though it remained below the 0.90 benchmark, highlighting sensitivity to sample size [52].

Despite these improvements, the CFI and TLI remain slightly below the 0.95 benchmark, suggesting the need for further refinements. Given the complexity of the Worker’s WellBQ, employing flexible cutoffs—which account for sample size, degrees of freedom, and model complexity—may provide a more accurate fit evaluation[53]. For instance, less stringent CFI thresholds could better accommodate model complexity, while adjusting RMSEA criteria could prevent the rejection of valid structures.

Future refinements should focus on enhancing CFI and TLI, optimizing item loadings, and increasing sample size while utilizing flexible cutoffs for improved assessment accuracy. Modification indices should be reviewed to refine the model, including allowing correlated residuals or removing weakly loading items, ensuring theoretical justification and construct validity [39,54]. This approach minimizes the risk of both false rejection of well-fitting models (Type I error) and false acceptance of misspecified models (Type II error), leading to a more robust evaluation framework. Compared to the Italian version of WellBQ [47], the Malay version excluded eight items (e.g., wage satisfaction, workplace flexibility) to improve model fit, reflecting cultural and occupational nuances, such as non-monetary job satisfaction and workplace flexibility expectations in Malaysia. These differences highlight the need for context-specific adjustments during validation.

Higher-order CFA streamlined the model by consolidating first-order factors (e.g., job engagement, workplace safety) under broader constructs (e.g., workplace evaluation, workplace culture). This hierarchical approach enhances the tool’s usability for addressing complex occupational well-being determinants [39].

The validated Malay WellBQ comprises 109 items across five major domains: Work Evaluation and Experience, Workplace Policies and Culture, Workplace Physical Environment and Safety Climate, Health Status, and Home, Community, and Society. The “Workplace Policies and Culture” domain demonstrated strong reliability and effectively captured organizational dynamics in Malaysia, while the “Health Status” domain addressed mental health and productivity factors, aligning with the original NIOSH WellBQ and its Italian adaptation [47].

However, the “Home, Community, and Society” domain exhibited lower AVE scores, indicating potential challenges in fully capturing these constructs within the Malaysian context. Although discriminant validity was achieved within domains, issues arose between some of the five major domains, reflecting interconnectedness such as the relationship between workplace policies, safety, and health. These findings emphasize the need for ongoing refinement to ensure the comprehensive representation of worker well-being across diverse occupational settings.

### Implications and applications

The validated Malay Worker’s Well-Being Questionnaire (WellBQ) offers a robust, multidimensional framework to assess and enhance workforce well-being in Malaysia. Prior research highlights the critical role of well-being assessments in shaping occupational health interventions [55–59]. By addressing physical, mental, emotional, and social dimensions, the Malay WellBQ provides a data-driven tool for identifying workplace resource gaps and implementing targeted strategies [60].

Rooted in the biopsychosocial model [20], the Malay WellBQ extends beyond traditional biomedical perspectives by capturing the complex determinants of worker well-being across diverse occupational settings. It encompasses key domains such as workplace safety, work-life balance, and community engagement, enabling organizations to develop comprehensive policies that enhance resilience, productivity, and overall health outcomes [60]. Moreover, by acknowledging the interconnectedness of work and life, the Malay WellBQ integrates factors beyond the workplace—including home, community, and societal influences [61]—to foster a holistic approach to workforce well-being.

This broader perspective supports evidence-based policy development that aligns with both global well-being frameworks and local workplace contexts. Consistent with research on supportive work environments and employee retention [56,58,59,62], the Malay WellBQ equips leaders with actionable insights to refine workplace policies, create supportive environments, and enhance employee satisfaction. Future studies should examine its longitudinal impact across industries to further validate its role in shaping occupational health and HR strategies.

### Challenges and limitations

The study faced several limitations that warrant consideration. Despite a high response rate (81.9%), funding constraints limited the use of incentives, which may have affected participation. Research suggests that incentives can enhance survey response rates, particularly in healthcare studies [63]. However, ethical concerns arise regarding undue influence, fairness, and recruitment bias, especially when monetary incentives disproportionately attract individuals from lower socioeconomic backgrounds [64,65]. To ensure ethical and equitable research participation, payment frameworks should distinguish between reimbursement, compensation, and incentives to prevent coercion and uphold fairness [66]. Alternative engagement strategies, such as institutional support, flexible survey administration, and non-monetary incentives, can enhance participation while maintaining voluntary consent. Ensuring that payments remain proportionate to time and effort is essential to balancing ethical considerations and study feasibility.

This study was conducted within a single hospital, which may limit the generalizability of its findings. While this setting provides valuable insights and allows for efficient use of resources by minimizing logistical and coordination challenges, the results may not fully capture the diversity of broader healthcare settings [67]. However, to enhance external validity and applicability, future research should include diverse samples from multiple hospitals and regions, ensuring a broader representation of the target population [68–70].

Furthermore, the study’s exclusive focus on the healthcare sector limits its generalizability to other industries with distinct workplace dynamics, such as manufacturing or education. Challenges in convergent validity were also noted, with some Average Variance Extracted (AVE) values falling below the recommended threshold, indicating gaps in capturing the underlying constructs of certain domains, despite adequate composite reliability (CR). Lastly, the limited involvement of stakeholders, such as policymakers and workers from diverse industries, may have constrained the broader applicability of the findings, highlighting the need for more inclusive engagement in future studies.

### Recommendations

To enhance the reliability and validity of future measurement models, increasing the sample size is crucial for robust results. Future studies may consider conducting Exploratory Factor Analysis (EFA) before Confirmatory Factor Analysis (CFA) to examine factor structures, particularly when applying a questionnaire to a new cultural or demographic group or modifying its items. Cultural differences can influence how constructs are perceived, and EFA helps ensure the reliability and relevance of adapted measures. Studies like the validation of the East Asian Acculturation Measure (EAAM) [71]and the Composite Physical Function (CPF) scale [72] highlight EFA’s role in refining instruments. Experts emphasize that CFA alone may not detect emerging or missing factor loadings, making EFA a crucial step before model confirmation. IBM SPSS Amos has limitations in handling categorical variables, which may affect model estimation accuracy [73]. Alternative software, such as Mplus (which supports robust weighted least squares estimation) or R’s lavaan package, provides greater flexibility in structural equation modeling [74,75]. Future research should consider these tools for improved model estimation and handling of categorical responses. Test-retest reliability and comparisons with other well-being instruments should also be explored to establish theoretical validity. Additionally, assessing concurrent validity should be prioritized to determine how well the Malay WellBQ aligns with other established measures of worker well-being. By engaging a broader range of stakeholders, including policymakers, industry leaders, and representatives from underrepresented sectors, could further enhance the applicability and impact of the Malay WellBQ.

## Conclusion

The final validated Malay WellBQ is a robust tool for assessing worker well-being in Malaysia, offering a multidimensional framework that integrates culturally relevant constructs with international standards. Despite challenges in item relevance and model fit, strategic adjustments ensured the tool’s reliability and validity. By providing comprehensive insights into worker well-being, the Malay WellBQ has significant potential to inform policies and interventions that promote health, productivity, and resilience in diverse occupational settings.

## Supporting information

S1 Worker WellBQ (Malay version). (DOCX)

S2 Dataset CFA (WellBQ). (SAV)

S3 NIOSH Worker WellBQ (English version). (PDF)
