# Supplementary material for: Translation and validation of Malay version of NIOSH worker well-being questionnaire (WellBQ)
Source: PLoS One. 2025 May 9;20(5):e0322451. doi: 10.1371/journal.pone.0322451 (PMC12064195; doi:10.1371/journal.pone.0322451)

**BORANG SOAL SELIDIK KESEJAHTERAAN PEKERJA (WellBQ)**

Nionella Bt Stephen Sampil, MPH, MD

Aziah bt Daud, M COMM MED, MD, B MED SC

Suhaily bt Mohd Hairon, PhD, MPH, MBBS

**MAKLUMAT ASAS PESERTA (Bahagian A)**

1. **Umur (sila pilih linkungan umur di bawah)**

- 18 - 29 tahun
- 30 - 44 tahun
- 45 – 60 tahun

1. **Jantina**

- Lelaki
- Wanita

1. **Bangsa**

- Melayu
- Cina
- India
- Lain-lain bumiputera

1. **Pekerjaan**

- Doktor Pakar
- Pegawai Perubatan (Medical Officer)
- Pegawai Perubtan Siswazah (Houseman)
- Pegawai Pergigian
- Pegawai Farmasi
- Penolong Pegawai Farmasi
- Penolong Pegawai Perubatan
- Pegawai Sains Makmal
- Juruteknik Makmal Perubatan
- Jururawat
- Atendan/Pembantu Perawatan Kesihatan

1. **Jenis Pekerjaan**

- Pekerjaan secara tetap
- Pekerjaan secara Kontrak

1. **Tahap Pendidikan**

- Sekolah rendah
- Sekolah menengah
- Diploma
- Ijazah Sarjana Muda dan tahap lebih tinggi

1. **Berapa tempoh masa anda telah bekerja (tahun)?**

**(Sila pilih jawapan di bawah)**

- Kurang setahun
- 1 – 10 tahun
- 11 -20 tahun
- Lebih 20 tahun

**BAHAGIAN B: Borang Soal Selidik WellBQ**

**DOMAIN 1: PENILAIAN PEKERJAAN DAN PENGALAMAN**

Soalan dalam bahagian ini menanyakan perasaan anda tentang pelbagai aspek pekerjaan anda.Jika awak mempunyai lebih daripada satu pekerjaan, sila jawab soalan berkaitan dengan pekerjaan utama anda.

**S1. Secara keseluruhan, saya ____ dengan pekerjaan saya.**

- Tidak berpuas hati
- Kurang berpuas hati
- Agak Berpuas hati
- Sangat berpuas hati

**S2. Saya ____ dengan gaji saya.**

- Tidak berpuas hati
- Kurang berpuas hati
- Agak Berpuas hati
- Sangat berpuas hati

**S3.  Saya ____ dengan  faedah pekerja yang disediakan oleh majikan saya. (Contoh: insurans kesihatan, bonus tahunan,cuti tahunan, kursus tahunan berbayar)**

- Tidak berpuas hati
- Kurang berpuas hati
- Agak Berpuas hati
- Sangat berpuas hati
- Tidak berkaitan

**S4. Saya ____ dengan peluang yang diberikan oleh majikan untuk memajukan diri dalam pekerjaan.**

- Tidak berpuas hati
- Kurang berpuas hati
- Agak Berpuas hati
- Sangat berpuas hati

**S5. Saya boleh mengharapkan sokongan kerja dari rakan sekerja saya apabila saya memerlukannya.**

- Sangat tidak setuju
- Tidak setuju
- Agak Setuju
- Sangat setuju
- Tidak berkaitan

**S6. Saya rasa pekerjaan saya terjamin (jaminan pekerjaan yang tetap)**

- Sangat tidak setuju
- Tidak setuju
- Agak Setuju
- Sangat setuju
- Tidak berkaitan

**S7. Kerja yang saya lakukan adalah bermakna bagi saya.**

- Sangat tidak setuju
- Tidak setuju
- Agak Setuju
- Sangat setuju

**S8. Kerja yang saya lakukan mempunyai tujuan yang lebih tinggi.**

- Sangat tidak setuju
- Tidak setuju
- Agak Setuju
- Sangat setuju

**S9. Berapa kerap anda mengalami perasaan ini semasa anda bekerja? (Tandakan (√) pada bahagian yang berkenaan)**

|  | Tidak pernah | Hampir tidak pernah (beberapa kali setahun atau kurang) | Jarang-jarang (sekali sebulan atau kurang) | Kadang-kadang (beberapa kali sebulan) | Selalunya (sekali seminggu) | Sangat kerap (beberapa kali seminggu) | Sentiasa (setiap hari) |
| --- | --- | --- | --- | --- | --- | --- | --- |
| A. Bersemangat |  |  |  |  |  |  |  |
| B. Bertenaga |  |  |  |  |  |  |  |
| C. Puas hati |  |  |  |  |  |  |  |
| D. Tenang |  |  |  |  |  |  |  |
| E. Gelisah |  |  |  |  |  |  |  |
| F. Marah |  |  |  |  |  |  |  |
| G. Muram |  |  |  |  |  |  |  |
| H. Patah semangat |  |  |  |  |  |  |  |

**S10. Berapa kerapkah anda mengalami gejala keletihan atau kelesuan semasa bekerja?**

- Tidak pernah
- Hampir tidak pernah (beberapa kali setahun atau kurang)
- Jarang – jarang (sekali sebulan atau kurang)
- Kadang-kadang (beberapa kali sebulan)
- Kerap (sekali seminggu)
- Sangat kerap (beberapa kali seminggu)
- Sentiasa (setiap hari)

**S11.  Kerja saya memberi inspirasi atau motivasi kepada saya.**

- Tidak pernah
- Hampir tidak pernah (beberapa kali setahun atau kurang)
- Jarang – jarang (sekali sebulan atau kurang)
- Kadang-kadang (beberapa kali sebulan)
- Kerap (sekali seminggu)
- Sangat kerap (beberapa kali seminggu)
- Sentiasa (setiap hari)

**S12.  Saya terlalu fokus dalam kerja saya.**

- Tidak pernah
- Hampir tidak pernah (beberapa kali setahun atau kurang)
- Jarang – jarang (sekali sebulan atau kurang)
- Kadang-kadang (beberapa kali sebulan)
- Kerap (sekali seminggu)
- Sangat kerap (beberapa kali seminggu)
- Sentiasa (setiap hari)

**S13.   Saya merasakan keinginan untuk datang bekerja setiap hari.**

- Tidak pernah
- Hampir tidak pernah (beberapa kali setahun atau kurang)
- Jarang – jarang (sekali sebulan atau kurang)
- Kadang-kadang (beberapa kali sebulan)
- Kerap (sekali seminggu)
- Sangat kerap (beberapa kali seminggu)
- Sentiasa (setiap hari)

**DOMAIN 2: DASAR DAN BUDAYA TEMPAT KERJA**

Soalan dalam bahagian ini bertanyakan perasaan anda tentang organisasi anda dan tentang faedah dan program kesihatan yang terdapat di tempat kerja. Jika anda mempunyai lebih daripada satu kerja, sila jawab soalan berdasarkan pekerjaan utama anda.

**S14. Di organisasi saya, saya dilayan dengan hormat dan baik.**

- Sangat tidak setuju
- Tidak setuju
- Agak Setuju
- Sangat setuju
- Tidak berkaitan

**S15.  Organisasi saya menghargai sumbangan saya.**

- Sangat tidak setuju
- Tidak setuju
- Agak Setuju
- Sangat setuju
- Tidak berkaitan

**S16.  Organisasi saya mengambil berat tentang kepuasan saya di tempat kerja.**

- Sangat tidak setuju
- Tidak setuju
- Agak Setuju
- Sangat setuju
- Tidak berkaitan

**S17.  Organisasi saya bersedia untuk memberi sokongan untuk membantu saya melaksanakan tugas saya dengan sebaik mungkin.**

- Sangat tidak setuju
- Tidak setuju
- Agak Setuju
- Sangat setuju
- Tidak berkaitan

**S18. Saya menerima pengiktirafan untuk kerja yang dilakukan dengan baik.**

- Sangat tidak setuju
- Tidak setuju
- Agak Setuju
- Sangat setuju

**S19. Saya diberi kebebasan dalam melaksanakan pekerjaan saya.**

- Sangat tidak setuju
- Tidak setuju
- Agak Setuju
- Sangat setuju

**S20.  Saya mempercayai pengurusan yang integriti di organisasi saya.**

- Sangat tidak setuju
- Tidak setuju
- Agak Setuju
- Sangat setuju
- Tidak berkaitan

**S21.   Organisasi saya komited terhadap kesihatan dan kesejahteraan pekerja.**

- Sangat tidak setuju
- Tidak setuju
- Agak Setuju
- Sangat setuju
- Tidak berkaitan

**S22.   Organisasi saya menggalakkan dan menyediakan peluang untuk melibatkan diri dalam tingkah laku yang sihat, seperti aktif secara fizikal, mengamalkan pemakanan yang sihat, hidup bebas  dari rokok/ tembakau, dan menguruskan tekanan saya.**

- Sangat tidak setuju
- Tidak setuju
- Agak Setuju
- Sangat setuju
- Tidak berkaitan

**S23.  Adakah faedah berikut ditawarkan oleh majikan anda? (Tandakan (√) pada bahagian yang berkenaan)**

|  | Ya | Tidak | Tidak Tahu | Tidak berkenaan |
| --- | --- | --- | --- | --- |
| 1. Insurans kesihatan |  |  |  |  |
| 1. Bantuan pendidikan/tuisyen |  |  |  |  |
| 1. Persaraan (majikan menyumbang untuk simpanan bersara) |  |  |  |  |
| D. Cuti bersalin bergaji |  |  |  |  |
| E. Cuti isteri bersalin bergaji |  |  |  |  |
| F. Cuti sakit bergaji |  |  |  |  |
| G. Cuti menjaga orang lain bergaji (contohnya, menjaga ahli keluarga yang sakit) |  |  |  |  |
| H. Cuti hilang upaya bergaji |  |  |  |  |
| I. Cuti percutian bergaji |  |  |  |  |
| J. Lain- lain cuti bergaji (seperti cuti berkabung, kecemasan dan menjadi saksi di mahkamah) |  |  |  |  |
| K. Kebenaran mengambil cuti tanpa gaji |  |  |  |  |
| L. Pilihan transit (seperti bantuan pengangkutan pergi dan balik kerja) |  |  |  |  |
| M. Pemberian rawatan di tempat bekerja |  |  |  |  |
| N. Program bantuan pekerja (seperti program membantu pekerja berkaitan masalah peribadi atau yang berkaitan dengan kerja) |  |  |  |  |

**S24.   Adakah program atau perkhidmatan kesihatan dan kesejahteraan berikut tersedia untuk anda di tempat anda bekerja?**

|  | Ya | Tidak | Tidak Tahu | Tidak berkenaan |
| --- | --- | --- | --- | --- |
| A. Program pendidikan dan promosi kesihatan (program kesihatan) |  |  |  |  |
| B. Diskaun keahlian gim atau pusat kesihatan di tempat bekerja (termasuk gim dan/ atau ruang untuk kelas berkumpulan) |  |  |  |  |
| C. Ruang umum atau hub aktiviti (ruang untuk aktiviti berkumpulan seperti bersosial, kelas senaman, dll) |  |  |  |  |
| D. Program berhenti merokok |  |  |  |  |
| 1. Program pemulihan dadah dan alkohol |  |  |  |  |
| 1. Program pengurusan stress |  |  |  |  |
| 1. Akses kepada pilihan makanan dan snek yang sihat semasa waktu bekerja |  |  |  |  |

**S25.   Berapa kerapkah kehidupan peribadi anda terganggu atas sebab keperluan pekerjaan?**

- Tidak pernah
- Hampir tidak pernah (beberapa kali setahun atau kurang)
- Jarang – jarang (sekali sebulan atau kurang)
- Kadang-kadang (beberapa kali sebulan)
- Kerap (sekali seminggu)
- Sangat kerap (beberapa kali seminggu)
- Sentiasa (setiap hari)

**S26.  Berapa kerapkah pekerjaan anda terganggu atas sebab keperluan kehidupan peribadi?**

- Tidak pernah
- Hampir tidak pernah (beberapa kali setahun atau kurang)
- Jarang – jarang (sekali sebulan atau kurang)
- Kadang-kadang (beberapa kali sebulan)
- Kerap (sekali seminggu)
- Sangat kerap (beberapa kali seminggu)
- Sentiasa (setiap hari)

**S27. Saya mempunyai kebebasan untuk bekerja di pejabat atau dari rumah.**

- Sangat tidak setuju
- Tidak setuju
- Agak Setuju
- Sangat setuju
- Tidak berkaitan

**DOMAIN 3: PERSEKITARAN FIZIKAL DAN KESELAMATAN TEMPAT KERJA**

Soalan dalam bahagian ini bertanya tentang ciri fizikal persekitaran kerja anda dan keadaan keselamatan di tempat anda bekerja. Jika anda mempunyai lebih daripada satu kerja, sila jawab soalan berdasarkan pekerjaan utama anda.

**S28.  Secara keseluruhan, sejauh manakah anda rasa tempat kerja anda selamat?**

- Sangat tidak selamat
- Tidak selamat
- Agak Selamat
- Sangat selamat

**S29.    Sila nyatakan sejauh mana anda bersetuju atau tidak bersetuju dengan setiap pernyataan berikut tentang amalan keselamatan di tempat kerja anda. (Tandakan (√) pada bahagian yang berkenaan)**

|  | Sangat tidak setuju | Agak tidak setuju | Agak setuju | Sangat setuju | Tidak berkaitan |
| --- | --- | --- | --- | --- | --- |
| A. Pihak pengurusan bertindak balas dengan cepat untuk menyelesaikan masalah apabila diberitahu tentang ancaman keselamatan. |  |  |  |  |  |
| B. Pihak pengurusan menitikberatkan pemeriksaan dan audit keselamatan menyeluruh dan dilaksanakan secara berterusan. |  |  |  |  |  |
| C. Pihak pengurusan menyediakan semua peralatan yang diperlukan untuk menjalankan kerja dengan selamat. |  |  |  |  |  |
| D. Pihak pengurusan melaburkan banyak masa dan wang dalam memberi latihan keselamatan untuk pekerja. |  |  |  |  |  |
| E.Pihak pengurusan mendengar dengan teliti idea pekerja tentang meningkatkan keselamatan. |  |  |  |  |  |
| F. Pihak pengurusan memberi kuasa kepada pegawai keselamatan dalam  melakukan tugas mereka. |  |  |  |  |  |

**S30.     Berkaitan dengan pekerjaan saya sekarang, inilah perasaan saya tentang topik berikut: (Tandakan (√) pada bahagian yang berkenaan)**

|  | Tidak sama sekali berpuas hati | Tidak terlalu berpuas hati | Agak berpuas hati | Sangat Berpuas Hati |
| --- | --- | --- | --- | --- |
| A. Keadaan persekitaran (suhu bilik yang sesuai, pencahayaan, pengudaraan) |  |  |  |  |
| B. Persekitaran fizikal (contohnya, infrastruktur bangunan, susun atur kawasan kerja (ergonomik), reka bentuk) |  |  |  |  |
| C. Persekitaran kerja yang menyenangkan (contohnya, keharmonian antara rakan sekerja, majikan) |  |  |  |  |

**S31.  Kemudahan untuk orang kurang upaya dan/atau berkeperluan khas (laluan untuk kerusi roda, bilik laktasi)**

- Tidak berpuas hati
- Kurang berpuas hati
- Agak Berpuas hati
- Sangat berpuas hati
- Tidak berkaitan

**S32.  Saya merasa didiskriminasi dalam pekerjaan kerana usia saya.**

- Sangat tidak setuju
- Tidak setuju
- Agak Setuju
- Sangat setuju

**S33. Saya merasa didiskriminasi dalam pekerjaan saya kerana bangsa atau asal usul etnik saya.**

- Sangat tidak setuju
- Tidak setuju
- Agak Setuju
- Sangat setuju

**S34.  Saya merasa didiskriminasi dalam pekerjaan saya kerana jantina saya.**

- Sangat tidak setuju
- Tidak setuju
- Agak Setuju
- Sangat setuju

**S35.  Dalam tempoh 12 bulan yang lalu, adakah anda pernah terdedah dengan gangguan seksual semasa anda bekerja?**

- Ya
- Tidak

**S36. Dalam tempoh 12 bulan yang lalu, adakah anda terdedah kepada keganasan fizikal semasa anda bekerja?**

- Ya
- Tidak

**S37. Dalam tempoh 12 bulan yang lalu, adakah anda pernah dibuli, diancam atau diganggu dengan cara lain semasa anda bekerja?**

- Ya
- Tidak

**S38. Dalam tempoh 12 bulan yang lalu, pernahkah anda berada dalam situasi di mana pihak atasan anda atau rakan sekerja merendahkan anda atau, membuat kenyataan merendahkan anda, atau memanggil anda dalam istilah yang tidak profesional?**

- Ya
- Tidak
- Tidak terpakai

**DOMAIN 4: PERKARA MENGENAI STATUS KESIHATAN**

Soalan dalam bahagian ini bertanya tentang kesihatan fizikal dan mental anda serta tingkah laku berkaitan kesihatan.

**S39. Sekarang, fikirkan tentang kesihatan fizikal anda, termasuk penyakit fizikal dan kecederaan, dalam tempoh 30 hari lepas, berapa hari anda mengalami kesihatan fizikal yang tidak baik?**

**Masukkan bilangan hari (0–30)**

**S40.   Adakah anda pernah mengalami mana-mana yang berikut? (Tandakan (√) pada bahagian yang berkenaan)**

|  | Tidak pernah | Pada masa lalu | Ada pada masa kini |
| --- | --- | --- | --- |
| A. Artritis (radang sendi) |  |  |  |
| B. Gangguan penyakit radang lain (contohnya, sakit belakang, sakit leher, sakit lain) |  |  |  |
| C. Asma |  |  |  |
| D. Penyakit paru-paru, selain asma (contohnya, penyakit paru-paru kronik [COPD], bronkitis kronik, paru-paru bengkak (emfisema) |  |  |  |
| E. Kanser |  |  |  |
| F. Kemurungan |  |  |  |
| 1. Kencing Manis |  |  |  |
| H. Penyakit jantung |  |  |  |
| I. Darah tinggi |  |  |  |

**S41.   Adakah anda pernah mengalami insomnia (kesukaran tidur) yang kronik?**

- Tidak pernah
- Pada masa lalu
- Pada masa kini

**S42. Sekarang, fikirkan tentang kesihatan mental anda, dalam tempoh 30 hari lepas, berapa hari anda mengalami kesihatan mental yang tidak baik? (termasuk tekanan stres, kemurungan, kebimbangan, dan masalah dengan emosi)?**

**Masukkan bilangan hari (0–30)**

**S43.   Berapa kerapkah anda mengalami tekanan stres berkaitan topik berikut? (Tandakan (√) pada bahagian yang berkenaan)**

|  | Tidak pernah | Hampir tidak pernah (beberapa kali setahun atau kurang) | Jarang-jarang (sekali sebulan atau kurang) | Kadang-kadang (beberapa kali sebulan) | Selalunya (sekali seminggu) | Sangat kerap (beberapa kali seminggu) | Sentiasa (setiap hari) |
| --- | --- | --- | --- | --- | --- | --- | --- |
| A. Kesihatan anda |  |  |  |  |  |  |  |
| B. Kewangan anda |  |  |  |  |  |  |  |
| C. Perhubungan keluarga atau sosial anda |  |  |  |  |  |  |  |
| D. Kerja anda |  |  |  |  |  |  |  |

**S44.   Sepanjang 2 minggu yang lalu, berapa kerap anda berasa sedih, tertekan, atau putus asa?**

- Tiada
- Beberapa hari
- Kerap
- Hampir setiap hari

**S45.  Sepanjang 2 minggu yang lalu, berapa kerap anda rasa kurang minat atau keseronokan dalam melakukan sesuatu?**

- Tiada
- Beberapa hari
- Kerap
- Hampir setiap hari

**S46.   Sepanjang 2 minggu yang lalu, berapa kerap anda rasa gementar, cemas, atau terdesak?**

- Tiada
- Beberapa hari
- Kerap
- Hampir setiap hari

**S47.   Sepanjang 2 minggu lepas, berapa kerapkah anda rasa tidak dapat mengawal kebimbangan diri?**

- Tiada
- Beberapa hari
- Kerap
- Hampir setiap hari

**S48.   Dalam seminggu, berapa hari anda dapat melakukan sekurang-kurangnya 20 minit aktiviti fizikal intensiti yang tinggi? (Aktiviti intensiti tinggi berlangsung sekurang-kurangnya 10 minit dan meningkatkan kadar denyutan jantung anda, menjadikan anda berpeluh, dan mungkin membuat anda berasa sesak nafas; contohnya berlari, berbasikal pantas, mengangkat objek berat secara berterusan)**

**Masukkan bilangan hari (0–7)**

**S49.   Dalam seminggu, berapa hari anda dapat melakukan sekurang-kurangnya 30 minit aktiviti fizikal intensiti sederhana? (Aktiviti intensiti sederhana berlangsung sekurang-kurangnya 10 minit dan memerlukan lebih banyak usaha daripada yang diperlukan untuk tugas harian biasa; contohnya berjalan pantas, berkebun dan mengangkat objek ringan secara berterusan)**

**Masukkan bilangan hari (0–7)**

**S50.   Adakah anda menggunakan mana-mana produk tembakau berikut? (Tandakan (√) pada bahagian yang berkenaan)**

|  | Tidak pernah | Tidak lagi | Beberapa hari | Setiap hari |
| --- | --- | --- | --- | --- |
| A. Rokok |  |  |  |  |
| B. Cerut |  |  |  |  |
| C. Paip |  |  |  |  |
| D. Tembakau tanpa asap |  |  |  |  |
| E. Rokok elektronik |  |  |  |  |

**S51.   Berapa banyak minuman beralkohol yang anda minum dalam seminggu? (Satu minuman = satu bir, segelas wain, minuman keras atau minuman campuran.)**

**Masukkan bilangan minuman (Masukkan 0 jika tidak pernah minum)**

**S52.    Sepanjang tahun lalu, Jika anda adalah seorang lelaki, berapa kerap anda minum minuman beralkohol lebih daripada empat kali minuman alcohol?
Jika anda seorang Wanita, berapa kerap anda minum minuman beralkohol lebih daripada tiga kali pada mana-mana hari?**

**(Satu minuman = satu bir, segelas wain, minuman keras, atau minuman campuran)**

- Tidak pernah
- Sekali dalam 1 hari
- Beberapa kali dalam 2 atau 3 hari
- Kerap (lebih daripada 3 hari)

**S53.     Fikirkan tentang makanan yang merupakan sebahagian daripada diet biasa anda. Berapakah hidangan buah-buahan dan sayuran yang anda makan pada hari biasa?**

**(Satu hidangan adalah mana-mana yang berikut: 1 cawan sayur-sayuran berdaun mentah [kira-kira saiz genggaman tangan anda]; 1⁄2 cawan sayur-sayuran lain [dimasak atau mentah]; 1 keping buah bersaiz sederhana [kira-kira saiz besbol]; 1/2 cawan buah yang dicincang, dimasak atau dalam tin; atau 3⁄4 cawan jus sayuran atau buah-buahan.)**

- Kurang daripada 1 hidangan
- 1 hidangan
- 2 hidangan
- 3 hidangan
- 4 hidangan
- 5 atau lebih hidangan

**S54.      Berapa jam anda biasanya tidur pada waktu malam? Jika anda seorang pekerja syif, berapa jam anda dapat tidur dalam sehari?**

- 6 jam atau kurang
- 7 jam
- 8 jam
- 9 jam atau lebih

**S55.    Adakah anda terbatas dalam melakukan tugasan kerja kerana masalah fizikal, mental, atau emosi?**

- Tiada
- Sedikit
- Secara sederhana
- Amat sukar
- Tidak berkaitan/ tiada syarat

**S56.      Pada bulan lalu… (Tandakan (√) pada bahagian yang berkenaan)**

|  | Tidak pernah | Hampir tidak pernah  (satu masa sebulan) | Jarang-jarang (sekali seminggu atau kurang) | Kadang-kadang (beberapa kali seminggu) | Selalunya (sekali sehari) | Sangat kerap (beberapa kali satu hari) | Sentiasa  (setiap jam) |
| --- | --- | --- | --- | --- | --- | --- | --- |
| A. Berapa kerapkah anda tidak menumpukan perhatian yang cukup terhadap kerja anda? |  |  |  |  |  |  |  |
| B. Berapa kerapkah anda mendapati diri anda tidak berhati-hati bekerja seperti yang anda sepatutnya lakukan? |  |  |  |  |  |  |  |
| C. Berapa kerapkah anda tidak bekerja pada masa anda sepatutnya bekerja? |  |  |  |  |  |  |  |
| D. Berapa kerapkah anda kurang menyelesaikan tugasan berbanding pekerja lain? |  |  |  |  |  |  |  |

**S57.     Dalam tempoh 12 bulan yang lalu, adakah anda mengalami sebarang kecederaan yang berkaitan dengan kerja?**

- Ya
- Tidak

S58.     Jika anda mengalami sebarang kecederaan yang berkaitan dengan kerja dalam tempoh 12 bulan yang lalu, adakah ia memerlukan sebarang pertolongan cemas atau rawatan perubatan, perubahan dalam aktiviti pekerjaan, atau kurang masa bekerja?

- ya
- Tidak
- Tidak berkaitan/tidak mengalami kecederaan dalam tempoh 12 bulan yang lalu

**DOMAIN 5: RUMAH, KOMUNITI DAN MASYARAKAT**

Soalan dalam bahagian ini bertanya tentang pengalaman, perasaan dan aktiviti anda di luar waktu kerja.

**S59.      Secara umum, berapa kerap anda mengambil bahagian dalam mana-mana aktiviti berikut di luar waktu kerja? (Tandakan (√) pada bahagian yang berkenaan)**

|  | Tidak pernah | Hampir tidak pernah (beberapa kali setahun atau kurang) | Jarang-jarang (sekali sebulan atau kurang) | Kadang-kadang (beberapa kali sebulan) | Selalunya (sekali seminggu) | Sangat kerap (beberapa kali seminggu) | Sentiasa (setiap hari) | Tidak berkaitan |
| --- | --- | --- | --- | --- | --- | --- | --- | --- |
| 1. Aktiviti amal atau sukarelawan |  |  |  |  |  |  |  |  |
| 1. Aktiviti penjagaan domestik (contohnya, kanak-kanak, warga emas atau saudara/rakan yang kurang upaya, tetapi bukan sebagai sukarelawan atau di tempat amal) |  |  |  |  |  |  |  |  |
| 1. Tugas penyelenggaraan rumah (contohnya, memasak, pembersihan, pembaikan) |  |  |  |  |  |  |  |  |
| 1. Bergaul dengan rakan-rakan, keluarga, orang lain |  |  |  |  |  |  |  |  |
| 1. Mengambil latihan atau kursus pendidikan |  |  |  |  |  |  |  |  |
| 1. Aktiviti sukan, kebudayaan, atau aktiviti masa lapang |  |  |  |  |  |  |  |  |
| 1. Aktiviti santai atau aktiviti bersendirian yang terancang |  |  |  |  |  |  |  |  |

***Penghargaan***

Anda telah melengkapkan NIOSH WellBQ. Terima kasih kerana meluangkan masa anda!

Sebagai tanda penghargaan dalam meneyertai kajian ini, berikut merupakan info dan tips kesihatan yang anda boleh amalkan sewaktu bekerja.

**Panduan Aktif Sewaktu Hari Bekerja**


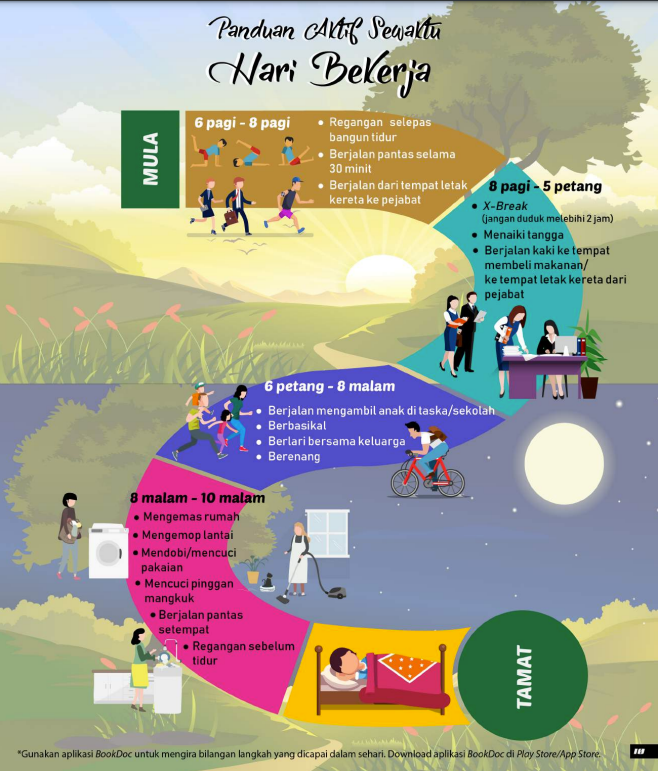

Supplement: S1 — (DOCX) [file pone.0322451.s001.docx]
