# Supplementary material for: Translation and validation of Malay version of NIOSH worker well-being questionnaire (WellBQ)
Source: PLoS One. 2025 May 9;20(5):e0322451. doi: 10.1371/journal.pone.0322451 (PMC12064195; doi:10.1371/journal.pone.0322451)
Supplement: S3 — (PDF) [file pone.0322451.s003.pdf]

NIOSH Worker Well-Being Questionnaire (WellBQ)

Centers for Disease Control and Prevention
National Institute for Occupational Safety and Health

NIOSH Worker Well-Being Questionnaire (WellBQ)

(Version 1)

Ramya Chari, PhD
Chia-Chia Chang, MPH, MBA
Steven L. Sauter, PhD
Elizabeth L. Petrun Sayers, PhD
Wenjing Huang, PhD
Gwenith G. Fisher, PhD

Department of Health and Human Services
Centers for Disease Control and Prevention
National Institute for Occupational Safety and Health

This document is in the public domain and may be freely copied or reprinted.

Disclaimer

Mention of any company or product does not constitute endorsement by the National Institute for Occupational Safety and Health (NIOSH), Centers for Disease Control and Prevention (CDC). In addition, citations of websites external to CDC/NIOSH do not constitute CDC/NIOSH endorsement of the sponsoring organizations or their programs or products. Furthermore, CDC/NIOSH is not responsible for the content of these websites. All web addresses referenced in this document were accessible as of the publication date.

Get More Information

Find NIOSH products and get answers to workplace safety and health questions:

1-800-CDC-INFO (1-800-232-4636) | TTY: 1-888-232-6348

CDC/NIOSH INFO: cdc.gov/info | cdc.gov/niosh

Monthly NIOSH eNews: cdc.gov/niosh/eNews

Suggested Citation

NIOSH [2021]. NIOSH worker well-being questionnaire (WellBQ). By Chari R, Chang C-C, Sauter SL, Petrun Sayers EL, Huang W, Fisher GG. Cincinnati, OH: U.S. Department of Health and Human Services, Centers for Disease Control and Prevention, National Institute for Occupational Safety and Health, DHHS (NIOSH) Publication No. 2021-110 (Revised 05/2024), https://doi.org/10.26616/NIOSHPUB2021110revised052024

DHHS (NIOSH) Publication No. 2021-110 (Revised 05/2024)

DOI: https://doi.org/10.26616/NIOSHPUB2021110revised052024

May 2024

This document was revised to make minor edits to formatting. No substantive changes were made to the information in this document

Table of Contents

Table of Contents 1
Acknowledgments 2
Overview of the NIOSH WellBQ 3
How the NIOSH WellBQ Can Be Used 3
Development of the NIOSH WellBQ 5
Content of the NIOSH WellBQ 6
Optional Items 7
Administering the NIOSH WellBQ 9
Data Privacy and Ethics 9
Item References and Permissions 10
Coding and Scoring Instructions 11
Data Interpretation 12
NIOSH WellBQ Codebook 13
NIOSH WellBQ 39
NIOSH WellBQ Item Source Table 60

Acknowledgments

Development of the National Institute for Occupational Safety and Health Worker Well-Being Questionnaire (NIOSH WellBQ) Version 1 was made possible through the time, expertise, and support provided by the research team listed below.

NIOSH
Steven L. Sauter, PhD
Chia-Chia Chang, MPH, MBA
L. Casey Chosewood, MD, MPH
Paul A. Schulte, PhD
Anita L. Schill, PhD, MPH, MA

The RAND Corporation
Ramya Chari, PhD
Elizabeth L. Petrun Sayers, PhD
Wenjing Huang, PhD
Vivian Towe, PhD
Lori Uscher-Pines, PhD
Jennifer Cerully, PhD

Colorado State University
Gwenith G. Fisher, PhD

NIOSH extends special thanks to the panel of experts who shared their expertise in the development of the worker well-being conceptual framework; the quality assurance review team at RAND; the NIOSH working group; and the external reviewers.

Overview of the NIOSH WellBQ

The National Institute for Occupational Safety and Health Worker Well-Being Questionnaire (NIOSH WellBQ) provides an integrated assessment of worker well-being across multiple spheres, including individuals' quality of working life, circumstances outside of work, and physical and mental health status. The questionnaire measures "worker" well-being as a holistic construct rather than simply "workplace" or "work-related" well-being. The NIOSH WellBQ is intended to help researchers, employers, workers, practitioners, and policymakers understand the well-being of workers and target interventions to improve worker well-being, among other applications.

The questionnaire comprises the five domains of worker well-being identified by NIOSH and the RAND Corporation in *Expanding the Paradigm of Occupational Safety and Health: A New Framework for Worker Well-being*[1]: (1) work evaluation and experience; (2) workplace policies and culture; (3) workplace physical environment and safety climate; (4) health status; and (5) home, community, and society. Please see that article for a discussion of the approach toward development of the worker well-being concept and the NIOSH WellBQ.

The five domains of worker well-being.

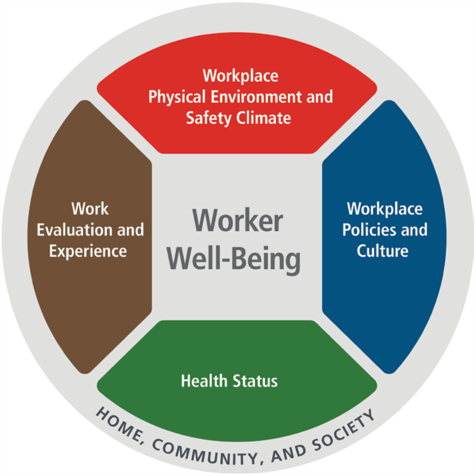

How the NIOSH WellBQ Can Be Used

The NIOSH WellBQ has many potential applications. It is designed to capture multiple facets of well-being to both broadly characterize the well-being of workers and inspect specific aspects of worker well-being. The questionnaire is designed to acquire data to develop a better understanding of the overall well-being of workers across the workforce as a whole, or within various worker subpopulations (e.g., among different occupational or industry sectors, at an organizational level, or among different demographic groupings of workers), and to identify aspects of worker well-being in need of special attention. As data are accumulated through widespread use of the NIOSH WellBQ in a diversity of settings, we anticipate that practitioners and policymakers will be able to establish benchmarks, norms, or targets for worker well-being across different working populations, occupations, and industries. The questionnaire may also

[1]Chari R, Chang CC, Sauter SL, Petrun Sayers EL, Cerully JL, Schulte P, Schill AL, Uscher-Pines L. [2018]. Expanding the paradigm of occupational safety and health: a new framework for worker well-being. J Occup Environ Med *60*(7):589–593.

be used for surveillance of changes in worker well-being in relation to economic conditions, societal trends, or changing governmental or organizational policies. Likewise, the NIOSH WellBQ can be used for applied research to investigate effects of deliberate interventions to influence worker well-being and associated outcomes, such as organizational performance, worker disability, and health care costs at the organizational or societal level.

Development of the NIOSH WellBQ

The NIOSH WellBQ was created by NIOSH and researchers at the RAND Corporation with funding from NIOSH. The design and content of the questionnaire were guided by a new conceptual framework that identifies the five domains of worker well-being. This framework was derived from an in-depth, multi-disciplinary literature review of well-being theories, research, and measurement tools by the RAND Corporation. Conditions of interest within each domain, which in turn defined the types of questions posed within each domain, were identified with assistance from a scientific panel with expertise in well-being and occupational safety and health.

The items in the questionnaire were strategically selected on the basis of their relevance to the five domains of worker well-being. Most of the items are adopted from existing instruments. After a draft questionnaire was created, cognitive testing was conducted on a convenience sample of employed individuals. Approval to field-test the questionnaire was then obtained from the Office of Management and Budget (OMB) on June 1, 2018 (OMB Control Number 0920-1234). The draft questionnaire was fielded on a probability-based sample by an internet-based survey administration service, following an initial pre-test of the questionnaire. Participants in the field test completed the draft questionnaire in approximately 20 minutes. Data from 975 respondents were analyzed to create multi-question scales, conduct factor analysis and other psychometric testing, conduct tests of concurrent validity, and eliminate less-productive items. Some items were revised to improve clarity, and a few items were eliminated from the piloted tool. The final questionnaire can be completed in approximately 15 minutes.

Content of the NIOSH WellBQ

The NIOSH WellBQ is designed to collect information across the five domains of worker well-being while minimizing burden to respondents. Because the questionnaire is meant to comprehensively assess worker well-being, NIOSH recommends administering the NIOSH WellBQ as a whole. Users who are interested in only specific topics may wish to administer other instruments, such as those listed in the NIOSH WellBQ Item Source Table.

The worker well-being topics covered by the questionnaire are detailed below.

NIOSH WellBQ Section 1. Work Evaluation and Experience (16 items)

- Job Satisfaction
- Wage Satisfaction
- Benefits Satisfaction
- Advancement Satisfaction
- Supervisor Support
- Coworker Support
- Job Security
- Job Autonomy
- Time Paucity/Work Overload
- Meaningful Work
- Work-related Positive Affect
- Work-related Negative Affect
- Work-related Fatigue
- Job Engagement

NIOSH WellBQ Section 2. Workplace Policies and Culture (14 items)

- Supportive Work Culture
- Management Trust
- Health Culture at Work
- Availability of Job Benefits
- Availability of Health Programs at Work
- Work to Non-work Conflict
- Non-work to Work Conflict
- Workplace/Schedule Flexibility

NIOSH WellBQ Section 3. Workplace Physical Environment and Safety Climate (10 items)

- Overall Workplace Safety
- Workplace Safety Climate
- Physical Work Environment Satisfaction
- Discrimination

- Work-related Sexual Harassment
- Work-related Physical Violence
- Work-related Bullying

NIOSH WellBQ Section 4. Health Status (23 items)

- Overall Health
- Days of Poor Physical Health
- Chronic Health Conditions
- Insomnia
- Days of Poor Mental Health
- Overall Stress
- Poor Mental Health
- Physical Activity
- Tobacco Use
- Alcohol Consumption
- Risky Drinking
- Healthy Diet
- Sleep Hours
- Sleepy at Work
- Cognitive Functioning Limitations
- Work Limitations
- Productivity
- Work-related Injury
- Injury Consequence

NIOSH WellBQ Section 5. Home, Community, and Society (5 items)

- Life Satisfaction
- Financial Insecurity
- Support Outside of Work
- Activities Outside of Work

Optional Items

Complementing the NIOSH WellBQ items are 15 optional items regarding employment circumstances and worker demographics. These sample items were selected based on their common use in population and occupational health surveys. These items are intended mainly for use in research or surveillance applications but may be useful for practical applications within organizations. The items may be modified, substituted, supplemented, or excluded, depending on the circumstances.

It is recommended that these items be placed at the end, following the NIOSH WellBQ.

OPTIONAL: Employment Circumstances (5 items)

- Work Arrangement
- Work Status
- Job Tenure
- Occupation
- Industry or Business

OPTIONAL: Demographic Information (10 items)

- Age
- Education
- Ethnicity
- Race
- Sex
- Sexual Orientation
- Household Income
- Head of Household
- Marital Status
- Number of Dependents

Administering the NIOSH WellBQ

Data Privacy and Ethics

Whether using the NIOSH WellBQ for research, surveillance, or practical applications within organizations, steps should be taken to protect the anonymity of participants throughout the data collection, analysis, and reporting processes. Participant names or other potentially identifying information such as their computer IP (internet protocol) address should not be collected together with their NIOSH WellBQ responses. Protecting worker privacy is an essential element of a *Total Worker Health®* approach.

When using the NIOSH WellBQ for research or surveillance,

- Establish procedures to obtain informed consent from prospective participants.
- Consult with a relevant human subjects review body and obtain approval as needed to ensure the use of ethical procedures and the protection of participants' privacy.

When an organization uses the instrument for more practical purposes, including the optional demographic or employment items and their variants can be a concern. Responses to these items, individually or in combination, could lead to participant identification and breach of privacy if they are linked at the individual level to responses to the NIOSH WellBQ items. This applies to the NIOSH WellBQ injury items (Q62 and Q63) as well. If the interest is simply an overall or global assessment of well-being, organizations may want to consider if it is necessary to collect demographic, employment, or injury information.

In some cases, organizations may wish to dig deeper and explore worker well-being in relation to various organizational or worker characteristics. This would necessitate linking responses to the demographic and employment items or the NIOSH WellBQ injury items with responses to other NIOSH WellBQ items at the individual level.

To help minimize risk to privacy under these circumstances:

- Use a third party operating under the guidance of a human subjects review body to administer and conduct subgroup analyses.
- Ensure that the linked response data are inaccessible to the organization. Make sure that any participant subgroup under study is large enough to prevent identification of a subgroup member and their responses to NIOSH WellBQ items.

Instructions to Participants

Provide the following information to individuals when soliciting their participation in the NIOSH WellBQ:

- Purpose of administering the questionnaire
- Procedures involved
- Participation is voluntary
- Risks/benefits to completing the questionnaire
- Steps taken to protect the anonymity of respondents throughout the data collection, analysis, and reporting processes
- Compensation (if any)
- Right to withdraw
- A point of contact for questions or concerns

Sample narrative:
"Welcome! Thank you for agreeing to fill out the National Institute for Occupational Safety and Health (NIOSH) Worker Well-Being Questionnaire, which is known as the NIOSH WellBQ. This survey asks about aspects of your job, workplace, health, and life outside of work. The information will help provide a better understanding of how workers in your organization [Edit as needed according to the application of the questionnaire] are doing and identify ways to improve worker well-being. [As needed, modify language on risks/benefits and procedures according to the context of the questionnaire survey.]

Several steps will be taken to ensure your anonymity. For example, the questionnaire does not ask for your name. Also, the IP address (i.e., the internet address for your computer) will not be recorded. Further, the answers you give will be combined with the answers from many other people who are taking the survey. [As needed, modify language on the protection of anonymity according to procedures and data management specific to the context of the questionnaire survey.]

You can choose not to participate. On any question, you can choose not to answer. You can stop answering questions and withdraw from participation at any time. In this case, none of your responses will be recorded. There are no right or wrong answers. Just base your answers on what you think. Some questions might not apply to your situation. In these cases, you can choose 'Does not apply' if it is one of the answer choices. If you have more than one job, please answer questions as they apply to your main job.

Please try to complete the survey in one sitting. It will take about 15 minutes to complete.

If you want to talk to someone about questions or concerns later, please contact [Fill in contact information for the responsible party within the organization]."

Item References and Permissions

This instrument may be freely reproduced, reprinted, or distributed. Many of the items have been adopted, with permission when necessary, from existing questionnaires. NIOSH obtained permission for using items for the purpose of this instrument. Permissions to adopt some items for this instrument do not allow fees to be charged for their use. The codebook provides specific use stipulations for each item when necessary. Please contact NIOSH *Total Worker Health* staff at twh@cdc.gov if you have questions.

Please see the NIOSH WellBQ Item Source Table for the full list of sources for questionnaire items.

Items that were drawn or adapted from the public domain or that were created by the research team are denoted as such in the codebook and the NIOSH WellBQ Item Source Table; no restrictions apply to the use of these items. Please note the suggested citation for the NIOSH WellBQ on page ii of this document.

Coding and Scoring Instructions

Single-question measures are coded according to the value associated with each response option, or as specified for open data fields. Multi-question scales are scored as specified in the codebook in *italics* (i.e., by calculating the sum or average of individual question values). Suggested variable names are also given for each question (indicated in bold **brown**) as well as names for scales (in bold **blue**) for use when creating data files. The responses "*Don't know*" and "*Does not apply*" should be coded as -88 and -99, respectively, to distinguish these from one another and from applicable responses, although users may wish to adopt a different coding convention for these two responses (e.g., -8 and -9).

Data Interpretation

The NIOSH WellBQ is a new instrument whose qualities are supported by extensive psychometric analyses based upon a large pilot study. As with all new instruments, the NIOSH WellBQ has limitations at this stage of development. The limitations will be addressed through accumulation of data and development of information in the future. For example, the available information is not sufficient to establish norms for measures across worker populations and industry and occupational sectors. Similarly, the available information is not sufficient to develop algorithms for creation of summary scores to characterize worker well-being. Applying the NIOSH WellBQ in a variety of workplace settings and among various worker populations will provide the information needed to address these limitations.

In the meantime, inferences regarding the status of worker well-being, intervention needs, intervention effects, etc., can be drawn from review of responses to questions and scale scores and from profiles of these values and scores across NIOSH WellBQ measures of interest. For immediate applications of the NIOSH WellBQ, please see the earlier discussion on "How the NIOSH WellBQ Can Be Used." In summary, users may apply the NIOSH WellBQ for the following purposes:

- To set benchmarks internal to an organization or workforce
- To examine changes over time or assess the impact of interventions
- To compare results between groups within the same facility or workforce or across organizations or working populations.

It is important to note, however, that the design of the NIOSH WellBQ does not permit absolute or clinical judgements of worker well-being, nor are there firm thresholds for scores that would signal actions to affect worker well-being.

Please see the NIOSH WellBQ webpage for resources that may be useful for improving worker well-being on the basis of applying the instrument. For guidance on development of actions or interventions after using the NIOSH WellBQ, please see Fundamentals of *Total Worker Health* Approaches.

NIOSH will develop further guidance for scoring and interpretation as new analysis arises from further research. Please check the NIOSH WellBQ webpage for updates.

NIOSH WellBQ Codebook

Section 1: Work Evaluation and Experience

The questions in this section ask how you feel about different aspects of your job. If you have more than one job, please answer questions as they apply to your *main* job.

Job Satisfaction JOBSAT

Q1. Overall, I am ____ with my job.

1 Not at all satisfied
2 Not too satisfied
3 Somewhat satisfied
4 Very satisfied

Item adapted from public domain source.

Wage Satisfaction WAGESAT

Q2. I am ____ with my wages.

1 Not at all satisfied
2 Not too satisfied
3 Somewhat satisfied
4 Very satisfied

**Item adapted from original wording for use in this questionnaire with permission from Dr. Scott Macdonald, with the provision that fees may not be charged for its use.*

Benefits Satisfaction BENSAT

Q3. I am ____ with the benefits provided by my employer.

1 Not at all satisfied
2 Not too satisfied
3 Somewhat satisfied
4 Very satisfied
-99 Does not apply

Item adapted from public domain source.

Advancement Satisfaction ADVNCSAT

Q4. I am ____ with my chances for advancement on the job.

1 Not at all satisfied
2 Not too satisfied
3 Somewhat satisfied
4 Very satisfied

**This item is adapted from the Minnesota Satisfaction Questionnaire (MSQ), from Vocational Psychology Research, University of Minnesota. The MSQ is available under a Creative Commons Attribution-NonCommercial 4.0 International License. The MSQ may be used for research or clinical work free of charge and without written consent, provided that you acknowledge Vocational Psychology Research, University of Minnesota, as the source of the material in your reproduced materials (printed or electronic). The license does not allow for commercial use or reproduction for sale. The MSQ may be used without cost, however, for employee surveys, provided that the survey is implemented within an organization and that no charges are*

made for its use. Please visit the following website to review specified conditions of free use for the MSQ in detail: http://vpr.psych.umn.edu/ All conditions of use for the MSQ apply to use of this item.

Supervisor Support SUPSUPP

Q5. I can count on my supervisor for support when I need it.

1 Strongly disagree
2 Somewhat disagree
3 Somewhat agree
4 Strongly agree
-99 Does not apply

Item adapted from public domain source.

Coworker Support COWSUPP

Q6. I can count on my coworkers for support when I need it.

1 Strongly disagree
2 Somewhat disagree
3 Somewhat agree
4 Strongly agree
-99 Does not apply

Item adapted from public domain source.

Job Security JOBSECUR

Q7. I feel my job is secure.

1 Strongly disagree
2 Somewhat disagree
3 Somewhat agree
4 Strongly agree

**Refer to permission details for item 2.*

Job Autonomy AUTONOMY

Q8. I am given a lot of freedom to decide how to do my own work.

1 Strongly disagree
2 Somewhat disagree
3 Somewhat agree
4 Strongly agree

Item drawn from public domain source.

Time Paucity/Work Overload TIMOVRLD

Q9. I never seem to have enough time to get everything done on my job.

1 Strongly disagree
2 Somewhat disagree
3 Somewhat agree
4 Strongly agree

Item adapted from public domain source.

Meaningful Work WKMEANAV

Calculate the average of the following two items (Q10, Q11):

Q10. The work I do is meaningful to me. **WKMEANG**

1 Strongly disagree
2 Somewhat disagree
3 Somewhat agree
4 Strongly agree

**Item adapted from original wording for use in this questionnaire with permission from Dr. Michael Steger, with the provision that fees may not be charged for its use.*

Q11. The work I do serves a greater purpose. **WKPURP**

1 Strongly disagree
2 Somewhat disagree
3 Somewhat agree
4 Strongly agree

**Refer to permission details for item 10.*

Work-related Positive Affect (POSAFFAV) and Work-related Negative Affect (NEGAFFAV)

Calculate the average of the four positive affect items (Q12A–12D).
Calculate the average of the four negative affect items (Q12E–12H).

Q12A–Q12H. How often do you experience these feelings when you are working?

	Never	Almost never (a few times a year or less)	Rarely (once a month or less)	Sometimes (a few times a month)	Often (once a week)	Very often (a few times a week)	Always (every day)
	1	2	3	4	5	6	7
**Work-related Positive Affect**							
A. Enthusiastic **POSENTHU**							
B. Energetic **POSENERG**							
C. Content **POSCONT**							
D. At ease **POSEASE**							
**Work-related Negative Affect**							
E. Anxious **NEGANXUS**							
F. Angry **NEGANGRY**							
G. Gloomy **NEGGLOOM**							
H. Discouraged **NEGDISCR**							

**These items are adapted from a copyrighted scale developed by Dr. Paul Spector. Dr. Spector has granted permission for use of these items in this questionnaire. However, Dr. Spector does not allow use of his scales for commercial purposes. Commercial use means you are charging someone a fee to provide a service that includes use of his scales. This restriction applies to these items. Please visit Dr. Spector's website (https://paulspector.com/.) to review conditions of free use in detail. Dr. Spector agreed to waive the requirement that data obtained from use of his scales in the NIOSH WellBQ be shared with him, but users are encouraged to voluntarily share data for these items with him when possible.*

Work-related Fatigue **WKFATIG**

Q13. How often do you experience fatigue when you are working?

1 Never
2 Almost never (a few times a year or less)
3 Rarely (once a month or less)
4 Sometimes (a few times a month)
5 Often (once a week)
6 Very often (a few times a week)
7 Always (every day)

**Refer to permission details for item 12.*

Job Engagement **ENGAGEAV**

Calculate the average of the following three items (Q14-16):

Q14. My work inspires me. **ENGINSPR**

1 Never
2 Almost never (a few times a year or less)
3 Rarely (once a month or less)
4 Sometimes (a few times a month)
5 Often (once a week)
6 Very often (a few times a week)
7 Always (every day)

**This item is adapted from a copyrighted scale developed by Dr. Wilmar Schaufeli. Dr. Schaufeli has granted permission for use of this item in this questionnaire. However, Dr. Schaufeli does not allow use of his scales for commercial purposes. Commercial use means you are charging someone a fee to provide a service that includes use of his scales. This restriction applies to this item. Please visit Dr. Schaufeli's website (https://www.wilmarschaufeli.nl/downloads/) to review conditions of free use in detail. Dr. Schaufeli agreed to waive the requirement that data obtained from use of his scales in the NIOSH WellBQ be shared with him, but users are encouraged to voluntarily share data for this item with him when possible.*

Q15. I am immersed in my work. **ENGIMMRS**

1 Never
2 Almost never (a few times a year or less)
3 Rarely (once a month or less)
4 Sometimes (a few times a month)
5 Often (once a week)
6 Very often (a few times a week)
7 Always (every day)

**Refer to permission details for item 14.*

Q16. When I get up in the morning, I feel like going to work. **ENGGOWRK**
1 Never
2 Almost never (a few times a year or less)
3 Rarely (once a month or less)
4 Sometimes (a few times a month)
5 Often (once a week)
6 Very often (a few times a week)
7 Always (every day)

**Refer to permission details for item 14.*

Section 2: Workplace Policies and Culture

The questions in this section ask how you feel about your organization and about benefits and health programs available at work. If you have more than one job, please answer questions as they apply to your *main* job.

Supportive Work Culture SUPCULAV

Calculate the average of the following five items (Q17-21):

Q17. At my organization, I am treated with respect. **SPCLRESP**

1 Strongly disagree
2 Somewhat disagree
3 Somewhat agree
4 Strongly agree
-99 Does not apply

Item adapted from public domain source.

Q18. My organization values my contributions. **SPCLEVAL**

1 Strongly disagree
2 Somewhat disagree
3 Somewhat agree
4 Strongly agree
-99 Does not apply

**Item adapted from original wording for use in this questionnaire with permission from Dr. Robert Eisenberger, with the provision that fees may not be charged for its use.*

Q19. My organization cares about my general satisfaction at work. **SPCLSAT**

1 Strongly disagree
2 Somewhat disagree
3 Somewhat agree
4 Strongly agree
-99 Does not apply

**Refer to permission details for item 18.*

Q20. My organization is willing to extend resources in order to help me perform my job to the best of my ability. **SPCLRESR**

1 Strongly disagree
2 Somewhat disagree
3 Somewhat agree
4 Strongly agree
-99 Does not apply

**Refer to permission details for item 18.*

Q21. I receive recognition for a job well done. **SPCLRECG**

1 Strongly disagree
2 Somewhat disagree
3 Somewhat agree
4 Strongly agree

**Refer to permission details for item 2.*

Management Trust MGMTTRST

Q22. I trust the management at my organization.

1 Strongly disagree
2 Somewhat disagree
3 Somewhat agree
4 Strongly agree
-99 Does not apply

Item adapted from public domain source.

Health Culture at Work HLCULAV

Calculate the average of the following two items (Q23, Q24):

Q23. My organization is committed to employee health and well-being. **HLCLCOMT**

1 Strongly disagree
2 Somewhat disagree
3 Somewhat agree
4 Strongly agree
-99 Does not apply

**Item adapted from original wording for use in this questionnaire with permission from Dr. Zandra Zweber, with the provision that fees may not be charged for its use.*

Q24. My organization encourages me and provides opportunities to engage in healthy behaviors, such as being physically active, eating a healthy diet, living tobacco free, and managing my stress. **HLCULBEH**

1 Strongly disagree
2 Somewhat disagree
3 Somewhat agree
4 Strongly agree
-99 Does not apply

**Refer to permission details for item 23.*

Availability of Job Benefits BENFSUM

Calculate the sum of the following 14 items (Q25A-25N):

Q25A–Q25N. Are the following benefits offered by your employer?

	Yes	No	Don't Know	Does not apply
	1	0	-88	-99
A. Health insurance **BENINSR**				
B. Assistance with education/tuition **BENEDUC**				
C. Retirement (employer contributions to retirement savings) **BENRETIR**				
D. Paid maternity leave **BENMATER**				
E. Paid paternity leave **BENPATER**				
F. Paid sick leave **BENSICKL**				
G. Other paid caregiving leave (for example, to care for sick family members) **BENCARGV**				
H. Paid disability leave **BENDISABL**				
I. Paid vacation days **BENVACAT**				
J. Other paid leave (for example, bereavement, emergency, jury duty) **BENOTHER**				
K. Ability to take unpaid leave **BENUNPAY**				
L. Transit options (such as help with transportation to and from work) **BENTRANS**				
M. On-site medical care **BENMEDIC**				
N. Employee assistance programs (such as programs that help workers with personal or work-related problems) **BENEAP**				

New items.

Availability of Health Programs at Work HLPGSUM

Calculate the sum of the following seven items (Q26A–26G):

Q26A–Q26G. Are the following health and wellness programs or services available to you at the place where you work?

	Yes	No	Don't know	Does not apply
	1	0	-88	-99
A. Health education and promotion programs (wellness programs) **HLPGEDUC**				
B. On-site fitness centers or gym membership discounts (includes a gym and/or space for group classes) **HLPGFIT**				
C. Common spaces or activity hubs (areas for group activities, such as socializing, exercise classes, etc.) **HLPGHUBS**				
D. Smoking cessation programs **HLPGSMOK**				
E. Alcohol and substance programs **HLPGALC**				
F. Stress management programs **HLPGSTRS**				
G. Access to healthy lunch and snack options **HLPGSNAC**				

New items.

Work to Non-work Conflict WNWCONF

Q27. How often do the demands of your job interfere with your personal life?

1 Never
2 Almost never (a few times a year or less)
3 Rarely (once a month or less)
4 Sometimes (a few times a month)
5 Often (once a week)
6 Very often (a few times a week)
7 Always (every day)

Item adapted from public domain source.

Non-work to Work Conflict NWWCONF

Q28. How often do the demands of your personal life interfere with your work on the job?

1 Never
2 Almost never (a few times a year or less)
3 Rarely (once a month or less)
4 Sometimes (a few times a month)
5 Often (once a week)
6 Very often (a few times a week)
7 Always (every day)

Item adapted from public domain source.

Workplace/Schedule Flexibility FLEXAV

Calculate the average of the following two items (Q29, Q30):

Q29. I have the freedom to vary my work schedule. **FLEXSCHD**

1 Strongly disagree
2 Somewhat disagree
3 Somewhat agree
4 Strongly agree

**Item adapted from original wording for use in this questionnaire with permission from Dr. Kristen M. Shockley, with the provision that fees may not be charged for its use.*

Q30. I have the freedom to work wherever is best for me—either at home or at my organization. **FLEXLOC**

1 Strongly disagree
2 Somewhat disagree
3 Somewhat agree
4 Strongly agree
-99 Does not apply

**Refer to permission details for item 29.*

Section 3: Workplace Physical Environment and Safety Climate

The questions in this section ask about physical characteristics of your work environment and safety conditions where you work. If you have more than one job, please answer questions as they apply to your *main* job.

Overall Workplace Safety SAFEOVER

Q31. Overall, how safe do you think your workplace is?

1 Very unsafe
2 Somewhat unsafe
3 Somewhat safe
4 Very safe

Item adapted from public domain source.

Workplace Safety Climate SAFCLMAV

Calculate the average of the following six items (Q32A-32F):

Q32A–Q32F. Please indicate how much you agree or disagree with each of the following statements about safety practices at your workplace.

	Strongly disagree	Somewhat disagree	Somewhat agree	Strongly agree	Does not apply
	1	2	3	4	-99
A. Management reacts quickly to solve the problem when told about safety hazards. **SCREACTS**					
B. Management insists on thorough and regular safety audits and inspections. **SCAUDITS**					
C. Management provides all the equipment needed to do the job safely. **SCEQUIPT**					
D. Management invests a lot of time and money in safety training for workers. **SCTRAING**					
E. Management listens carefully to workers' ideas about improving safety. **SCLISTEN**					
F. Management gives safety personnel the power they need to do their job. **SCPOWER**					

**Items are adapted from original wording for use in this questionnaire with permission from Dr. Dov Zohar, with the provision that fees may not be charged for their use.*

Physical Work Environment Satisfaction WRKENVAV

Calculate the average of the following four items (Q33A-33D):

Q33A–Q33C. On my present job, this is how I feel about the following topics:

	Not at all satisfied	Not too satisfied	Somewhat satisfied	Very satisfied
	1	2	3	4
A. The environmental conditions (heating, lighting, ventilation, etc.) **WKENCOND**				
B. The physical surroundings (for example, building infrastructure, work area layout, design) **WKENPHYS**				
C. The pleasantness of the work environment **WKENPLES**				

Q33D. The accommodations for disabilities and/or special needs (wheelchair ramps, lactation rooms, etc.) **WKENACOM**

1 Not at all satisfied
2 Not too satisfied
3 Somewhat satisfied
4 Very satisfied
-99 Does not apply

**For permission details for items 33A–33C, refer to item 4.*

Item 33D is new.

Discrimination DISCAV

Calculate the average of the following three items (Q34-36):

Q34. I feel discriminated against in my job because of my age. **DISCAGE**

1 Strongly disagree
2 Somewhat disagree
3 Somewhat agree
4 Strongly agree

Item adapted from public domain source.

Q35. I feel discriminated against in my job because of my race or ethnic origin. **DISCRACE**

1 Strongly disagree
2 Somewhat disagree
3 Somewhat agree
4 Strongly agree

Item adapted from public domain source.

Q36. I feel discriminated against in my job because of my gender. **DISCGEND**

1 Strongly disagree
2 Somewhat disagree
3 Somewhat agree
4 Strongly agree

Item adapted from public domain source.

Work-related Sexual Harassment SEXHARAS

Q37. In the past 12 months, were you sexually harassed by anyone while you were on the job?

1 Yes
0 No

Item adapted from public domain source.

Work-related Physical Violence PHYSVIOL

Q38. In the past 12 months, were you exposed to physical violence while you were on the job?

1 Yes
0 No

**This item is adapted from the Copenhagen psychosocial questionnaire – COPSOQ II. The COPSOQ is available under a Creative Commons Attribution-NonCommercial 4.0 International License. Under this license the COPSOQ is free to use if it is properly referenced and no fees are charged for its use. Please visit the following website to review specified conditions of free use for the COPSOQ in detail: https://nfa.dk/da/Vaerktoejer/Sporgeskemaer/Copenhagen-Psychosocial-Questionnaire-COPSOQ-II/Engelsk-udgave. All conditions of use for the COPSOQ II apply to use of this item.*

Work-related Bullying CONFLAV

Calculate the average of the following two items (Q39, Q40):

Q39. In the past 12 months, were you bullied, threatened, or harassed in any other way by anyone while you were on the job? **CONFBULY**

1 Yes
0 No

Item adapted from public domain source.

Q40. In the past 12 months, have you been in a situation where any of your superiors or coworkers put you down or were condescending to you, made demeaning remarks about you, or addressed you in unprofessional terms? **CONFPTDN**

1 Yes
0 No
-99 Does not apply

**Item adapted from original wording for use in this questionnaire with permission from Dr. Lilia Cortina and Dr. Vicki Magley, with the provision that fees may not be charged for its use.*

Section 4: Health Status

The questions in this section ask about your physical and mental health and health-related behaviors.

Overall Health HLTHOVER

Q41. Would you say that in general, your health is poor, fair, good, very good, or excellent?

1 Poor
2 Fair
3 Good
4 Very good
5 Excellent

Item adapted from public domain source.

Days of Poor Physical Health PHYSHLDY

Q42. Now, thinking about your physical health, which includes physical illness and injury, during the past 30 days, for how many days was your physical health not good?

Enter number of days (0–30) [ ]

Item adapted from public domain source.

Chronic Health Conditions HLTHSUM

Suggested scoring: Calculate the sum of the following nine items (Q43A–43I). Code Never as 0; code In the past and Have currently as 1.

Q43A–Q43I. Have you ever had any of the following?

	Never	In the past	Have currently
	0	1	2
A. Arthritis **HLTHARTH**			
B. Other musculoskeletal disorders (for example, back pain, neck pain, other pain) **HLTHMUSC**			
C. Asthma **HLTHASTH**			
D. Lung disease, other than asthma (for example, chronic obstructive pulmonary disease [COPD], chronic bronchitis, emphysema) **HLTHLUNG**			
E. Cancer **HLTHCANC**			
F. Depression **HLTHDEPR**			
G. Diabetes **HLTHDIAB**			
H. Heart disease **HLTHHRT**			
I. High blood pressure **HLTHBP**			

**Items 43A and 43C–43H are adapted from original wording for use in this questionnaire with permission from Dr. Karen Moseley, with the provision that fees may not be charged for their use.*

Items 43B and 43I are new.

Insomnia INSOMNIA

Suggested scoring: Code Never as 0; code In the past and Have currently as 1.

Q44. Have you ever had chronic insomnia?

0 Never
1 In the past
2 Have currently

**Refer to permission details for item 43A.*

Days of Poor Mental Health MENTHLDY

Q45. Now, thinking about your mental health, which includes stress, depression, anxiety, and problems with emotions, during the past 30 days, for how many days was your mental health not good?

Enter number of days (0–30)

Item adapted from public domain source.

Overall Stress STRSAV

Calculate the average of the following four items (Q46A-46D):

Q46A–Q46D. How often do you experience stress with regard to the following topics?

	Never	Almost never (a few times a year or less)	Rarely (once a month or less)	Sometimes (a few times a month)	Often (once a week)	Very often (a few times a week)	Always (every day)
	1	2	3	4	5	6	7
A. Your health **STRSHLTH**							
B. Your finances **STRSMONY**							
C. Your family or social relationships **STRSFMLY**							
D. Your work **STRSWORK**							

**Refer to permission details for item 43A.*

Poor Mental Health MNHLAV

Calculate the average of the following four items (Q47-50):

Q47. Over the last 2 weeks, how often have you been bothered by feeling down, depressed, or hopeless? **MNHLDOWN**

1 Not at all
2 Several days
3 More than half the days
4 Nearly every day

**Item drawn from the Patient Health Questionnaire for Depression and Anxiety (PHQ-4). The PHQ scales were developed by Drs. Robert L. Spitzer, Janet B.W. Williams, and Kurt Kroenke and colleagues. The PHQ scales are free to use and in the public domain.*

Q48. Over the last 2 weeks, how often have you been bothered by little interest or pleasure in doing things? **MNHLPLES**

1 Not at all
2 Several days
3 More than half the days
4 Nearly every day

**Refer to permission details for item 47.*

Q49. Over the last 2 weeks, how often have you been bothered by feeling nervous, anxious, or on edge? **MNHLNERV**

1 Not at all
2 Several days
3 More than half the days
4 Nearly every day

**Refer to permission details for item 47.*

Q50. Over the last 2 weeks, how often have you been bothered by not being able to stop or control worrying? **MNHLWORY**

1 Not at all
2 Several days
3 More than half the days
4 Nearly every day

**Refer to permission details for item 47.*

Physical Activity EXERAV

Calculate the average of the following two items (Q51, Q52):

Q51. In a typical week, how many days do you get at least 20 minutes of *high intensity* physical activity? (High intensity activity lasts at least 10 minutes and increases your heart rate, makes you sweat, and may make you feel out of breath; examples are running, fast cycling, and strenuous, continuous lifting of heavy objects.) **EXERVIG**

Enter number of days (0–7) [ ]

**Refer to permission details for item 43A.*

Q52. In a typical week, how many days do you get at least 30 minutes of *moderate intensity* physical activity? (Moderate intensity activity lasts at least 10 minutes and requires more effort than is needed for typical everyday tasks; examples are brisk walking, gardening, and continuous lifting of light objects.) **EXERMOD**

Enter number of days (0–7) [ ]

**Refer to permission details for item 43A.*

Tobacco Use TOBUSE

Suggested scoring: Calculate the sum of the following five items (Q53A–53E). Code Never and Not any more as 0; code Some days and Daily as 1.

Q53A-Q53E. Do you use any of the following tobacco products?

	Never	Not any more	Some days	Daily
	1	2	3	4
A. Cigarettes **SMOKCIGT**				
B. Cigars **SMOKCIGR**				
C. Pipes **SMOKPIPE**				
D. Smokeless tobacco **SMOKLESS**				
E. Electronic cigarettes **SMOKELEC**				

**Refer to permission details for item 43A.*

Alcohol Consumption ALCDRINK

Suggested scoring: For males, code more than 14 drinks as 1 and code 14 or fewer as 0. For females, code more than 7 drinks as 1 and code 7 or fewer as 0.

Q54. How many drinks of alcoholic beverages do you have in a typical week? (One drink = one beer, glass of wine, shot of liquor, or mixed drink.)

Enter number of drinks [ ]

**Refer to permission details for item 43A.*

Risky Drinking RSKDRINK

Suggested scoring: Code Never as 0 and the responses 2–4 as 1.

Q55. During the past year, how often have you had more than four drinks if you are a male, or more than three drinks if you are a female, on any single day? (One drink = one beer, glass of wine, shot of liquor, or mixed drink.)

1 Never
2 Once (1 day)
3 A few times (2-3 days)
4 Often (more than 3 days)

**Refer to permission details for item 43A.*

Healthy Diet NUTRITN

Q56. Think of the foods that are a part of your normal diet. How many servings of fruits and vegetables do you eat in a normal day?
(One serving is any of the following: 1 cup raw leafy greens [about the size of a small fist]; 1⁄2 cup of other vegetables [cooked or raw]; 1 medium piece of fruit [about the size of a baseball]; 1/2 cup chopped, cooked, or canned fruit; or 3⁄4 cup vegetable or fruit juice.)

1 Less than 1 serving
2 1 serving
3 2 servings
4 3 servings
5 4 servings
6 5 or more servings

**Refer to permission details for item 43A.*

Sleep Hours SLEEPQTY

Suggested scoring: Code 6 or fewer hours and 9 or more hours as 1; code 7 hours and 8 hours as 0.

Q57. How many hours of sleep do you usually get at night? If you are a shift worker, how many hours of sleep do you get a day?

1 6 or fewer hours
2 7 hours
3 8 hours
4 9 or more hours

**Refer to permission details for item 43A.*

Sleepy at Work WKSLEEPY

Q58. In the past 7 days, how often have you felt sleepy while at work?

1 Never
2 Rarely
3 Sometimes
4 Usually
5 Always

**Refer to permission details for item 43A.*

Cognitive Functioning Limitations COGLIM

Q59. Because of a physical, mental, or emotional condition, do you have serious difficulty concentrating, remembering, or making decisions?

1 Not at all
2 Slightly
3 Moderately
4 Extremely
-99 Does not apply/do not have condition

Item adapted from public domain source.

Work Limitations WORKLIM

Q60. Are you limited in the kind or amount of work you can do because of a physical, mental, or emotional problem?

1 Not at all
2 Slightly
3 Moderately
4 Extremely
-99 Does not apply/do not have problem

Item adapted from public domain source.

Productivity PRODAV

Calculate the average of the following four items (Q61A-61D):

Q61A–Q61D. In the past month...

	Never	Almost never (one time a month)	Rarely (once a week or less)	Sometimes (a few times a week)	Often (once a day)	Very often (a few times a day)	Always (every hour)
	1	2	3	4	5	6	7
A. How often did you not concentrate enough on your work? **PRODCONC**							
B. How often did you find yourself not working as carefully as you should? **PRODCARE**							
C. How often did you not work at times when you were supposed to be working? **PRODWORK**							
D. How often did you get less done than other workers? **PRODLESS**							

**Items are adapted from original wording for use in this questionnaire with permission from Dr. Ronald Kessler, with the provision that fees may not be charged for their use.*

Work-related Injury WKINJURY

Q62. During the past 12 months, did you experience any work-related injuries?
1 Yes
0 No

New item.

Injury Consequence INJEFFECT

Q63. If you experienced any work-related injuries in the past 12 months, did any of them require any first aid or medical treatment, change in job activities, or lost time from work?

1 Yes
0 No
-99 Does not apply/was not injured in the past 12 months

New item.

Section 5: Home, Community, and Society

The questions in this section ask about your experiences, feelings, and activities outside of work.

Life Satisfaction LIFESAT

Q64. In general, how satisfied are you with your life?

1 Not at all satisfied
2 Not too satisfied
3 Somewhat satisfied
4 Very satisfied

Item adapted from public domain source.

Financial Insecurity FINAV

Calculate the average of the following two items (Q65, Q66):

Q65. How worried are you right now about not being able to maintain the standard of living you enjoy? **FINSTDD**

1 Not worried at all
2 Not too worried
3 Moderately worried
4 Very worried

Item adapted from public domain source.

Q66. How worried are you right now about not having enough income to pay your normal monthly bills? **FINBILLS**

1 Not worried at all
2 Not too worried
3 Moderately worried
4 Very worried

Item adapted from public domain source.

Support Outside of Work NWKSUPP

Q67. How often do you get the social and emotional support you need from friends, family, or others outside of work?

1 Never
2 Rarely
3 Sometimes
4 Always

Item adapted from public domain source.

Activities Outside of Work NWKENSUM

Suggested scoring: Calculate the sum of the following seven items (Q68A-68G). Code Never (1) to Rarely (3) as 0; code Sometimes (4) to Always (7) as 1.

Q68A–Q68G. In general, how often do you take part in any of the following activities outside of work?

	Never	Almost never (a few times a year or less)	Rarely (once a month or less)	Sometimes (a few times a month)	Often (once a week)	Very often (a few times a week)	Always (every day)	Does not apply
	1	2	3	4	5	6	7	-99
A. Voluntary or charitable activities **NWKVOLUN**								
B. Domestic caregiving activities (for example, children, elderly or disabled relatives/ friends, but not in a volunteer or charity setting) **NWKCAREG**								
C. Home maintenance tasks (for example, cooking, cleaning, repairs) **NWKMAINT**								
D. Socializing with friends, family, others **NWKSOCIA**								

	Never	Almost never (a few times a year or less)	Rarely (once a month or less)	Sometimes (a few times a month)	Often (once a week)	Very often (a few times a week)	Always (every day)	Does not apply
E. Taking training or education courses **NWKTRAIN**								
F. Sporting, cultural, or leisure activities **NWKSPORT**								
G. Relaxation or planned solitary activities **NWKRELAX**								

**Items 68A, 68B, 68E, and 68F are adapted from the 6th European Working Conditions Survey (EWCS). Permission to use items from the EWCS is not necessary, as described on the survey website https://www.eurofound.europa.eu/sites/default/files/page/field_ef_documents/6th_ewcs_2015_final_source_master_questionnaire.pdf. However, published or disseminated materials containing these items must acknowledge the source of the data, and copies of this material must be shared with Sophia MacGoris (smg@eurofound.europa.eu) at the European Foundation for the Improvement of Living and Working Conditions, Wyattville Road, Loughlinstown, Co. Dublin, Ireland. Please visit the website listed above to review specified conditions of use in detail.*

Items 68C, 68D, and 68G are new.

Optional items

Employment Information

The questions in this section ask about your current working arrangements, occupation, and the industrial sector in which you are working. If you have more than one job, please answer questions as they apply to your *main* job.

Work Arrangement WKARRANG

E1. How would you describe your work arrangement in your job?

1 I am an independent contractor, an independent consultant, or a freelance worker.
2 I am on call and work only when called to work.
3 I am paid by a temporary agency.
4 I work for a contractor who provides workers and services to others under contract.
5 I am a regular, permanent employee.

Item drawn from public domain source.

Work Status WKSTATUS

E2. Is your job full-time or part-time?

1 Full-time
2 Part-time

New item.

Job Tenure JOBTEN

E3. How long have you worked in your job?

1 Less than 1 year
2 1–5 years
3 6–10 years
4 10–20 years
5 More than 20 years

New item.

Occupation OCCUP

E4. Select the occupation that best describes the kind of work you do in your job.

1 Architecture and Engineering
2 Arts, Design, Entertainment, Sports, and Media
3 Building and Grounds Cleaning and Maintenance
4 Business and Financial Operations
5 Computer and Mathematical
6 Community and Social Service
7 Construction and Extraction
8 Education Instruction and Library
9 Farming, Fishing, and Forestry
10 Food Preparation and Serving Related
11 Healthcare Practitioners and Technical
12 Healthcare Support
13 Installation, Maintenance, and Repair
14 Legal
15 Life, Physical, and Social Science
16 Management
17 Material Moving
18 Military Specific
19 Office and Administrative Support
20 Personal Care and Service
21 Production
22 Protective Service
23 Sales and Related
24 Transportation
25 Other (Please specify): ______________________________

Item adapted from public domain source.

Industry or Business INDUSTRY

E5. Select the kind of industry or business you work in for your job.

1 Arts, Entertainment, and Recreation
2 Accommodation and Food Services
3 Administrative and Support and Waste Management
4 Agriculture, Forestry, Fishing, and Hunting
5 Construction
6 Educational Services
7 Finance and Insurance
8 Health Care and Social Assistance
9 Information
10 Management of Companies and Enterprises
11 Manufacturing
12 Military
13 Mining, Quarrying, and Oil and Gas Extraction
14 Other Services, Except Public Administration
15 Public Administration
16 Professional, Scientific, and Technical Services
17 Real Estate and Rental and Leasing
18 Retail Trade
19 Transportation and Warehousing
20 Utilities
21 Wholesale Trade
22 Other (Please specify): ____________________________

Item drawn from public domain source.

Demographic Information

The questions in this section ask for basic information about yourself.

Age AGE

D1. What is your age?

1 18–29
2 30–44
3 45–64
4 65 and older

This is a NIOSH-suggested item and response options for collection of demographic data.

Education EDUC

D2. What is the highest level of school you have completed or the highest degree you have received?

1 Less than high school
2 High school/GED
3 Some college
4 Bachelor's degree or higher

This is a NIOSH-suggested item and response options for collection of demographic data.

Ethnicity ETHNICITY

D3. Do you consider yourself to be Hispanic or Latino?

1 Yes
2 No
7 Refused
9 Don't know

Item drawn from public domain source.

Race RACE

D4. What race or races do you consider yourself to be? Please select one or more of these categories.

1 White
2 Black/African American
3 American Indian
4 Alaska Native
5 Native Hawaiian
6 Other Pacific Islander
7 Asian
8 Some other race
97 Refused
99 Don't know

Item drawn from public domain source.

Sex SEX

D5. Are you male or female?

1 Male
2 Female
7 Refused
9 Don't know

Item drawn from public domain source.

Sexual Orientation SEXORI

D6. Do you think of yourself as gay/lesbian or gay; straight, that is, not gay/lesbian; bisexual; something else; or you don't know the answer?

1 Gay/lesbian
2 Straight, that is, not gay/lesbian
3 Bisexual
4 Something else
5 I don't know the answer
7 Refused
9 Don't know

Item drawn from public domain source.

Household Income HHINCOME

D7. What was your entire household income last year, before taxes?

1 <$20,000
2 $20,000 to $34,999
3 $35,000 to $49,999
4 $50,000 to $74,999
5 $75,000 to $99,999
6 $100,000 to $149,999
7 $150,000 to $199,999
8 $200,000 or more

This is a NIOSH-suggested item and response options for collection of demographic data.

Head of Household HHHEAD

D8. Are you the head of your household?

1 Yes
0 No

This is a NIOSH-suggested item and response options for collection of demographic data.

Marital Status MARSTAT

D9. What is your current marital status?

1 Married or living with partner
2 Widowed
3 Divorced
4 Separated
5 Never married

This is a NIOSH-suggested item and response options for collection of demographic data.

Dependents DEPEND

Calculate the sum of the following four items (D10A-D10D):

D10. How many dependents currently live in your household? Please enter the total number in each age category.

A. Total number of household members age 0 to 5 **NDEP05** ______

B. Total number of household members age 6 to 12 **NDEP612** ______

C. Total number of household members age 13 to 17 **NDEP1317** ______

D. Total number of household members age 18 or older **NDEP18UP** ______

These are NIOSH-suggested items and response options for collection of demographic data.

National Institute for Occupational Safety and Health
Worker Well-Being Questionnaire
(NIOSH WellBQ)
Version 1

Welcome! Thank you for agreeing to fill out the National Institute for Occupational Safety and Health Worker Well-Being Questionnaire, which is known as the NIOSH WellBQ. This survey asks about aspects of your job and workplace, your health, and your life outside of work. The information will help provide a better understanding of how workers in your organization are doing and identify ways to improve worker well-being.

You can choose not to participate. On any question, you can choose not to give an answer. There are no right or wrong answers. Just base your answers on what you think. Some questions might not apply to your situation. In these cases, you can choose "Does not apply" if it is one of the answer choices. Please try to complete the survey in one sitting. It will take about 15 minutes to complete.

If you have more than one job, please answer questions as they apply to your *main* job.

The questions in this section ask how you feel about different aspects of your job. If you have more than one job, please answer questions as they apply to your *main* job.

Q1. Overall, I am ____ with my job.

- ☐ Not at all satisfied
- ☐ Not too satisfied
- ☐ Somewhat satisfied
- ☐ Very satisfied

Q2. I am ____ with my wages.

- ☐ Not at all satisfied
- ☐ Not too satisfied
- ☐ Somewhat satisfied
- ☐ Very satisfied

Q3. I am ____ with the benefits provided by my employer.

- ☐ Not at all satisfied
- ☐ Not too satisfied
- ☐ Somewhat satisfied
- ☐ Very satisfied
- ☐ Does not apply

Q4. I am ____ with my chances for advancement on the job.

- ☐ Not at all satisfied
- ☐ Not too satisfied
- ☐ Somewhat satisfied
- ☐ Very satisfied

Q5. I can count on my supervisor for support when I need it.

- ☐ Strongly disagree
- ☐ Somewhat disagree
- ☐ Somewhat agree
- ☐ Strongly agree
- ☐ Does not apply

Q6. I can count on my coworkers for support when I need it.

- ☐ Strongly disagree
- ☐ Somewhat disagree
- ☐ Somewhat agree
- ☐ Strongly agree
- ☐ Does not apply

Q7. I feel my job is secure.

- ☐ Strongly disagree
- ☐ Somewhat disagree
- ☐ Somewhat agree
- ☐ Strongly agree

Q8. I am given a lot of freedom to decide how to do my own work.

- ☐ Strongly disagree
- ☐ Somewhat disagree
- ☐ Somewhat agree
- ☐ Strongly agree

Q9. I never seem to have enough time to get everything done on my job.

- ☐ Strongly disagree
- ☐ Somewhat disagree
- ☐ Somewhat agree
- ☐ Strongly agree

Q10. The work I do is meaningful to me.

- ☐ Strongly disagree
- ☐ Somewhat disagree
- ☐ Somewhat agree
- ☐ Strongly agree

Q11. The work I do serves a greater purpose.

- ☐ Strongly disagree
- ☐ Somewhat disagree
- ☐ Somewhat agree
- ☐ Strongly agree

Q12. How often do you experience these feelings when you are working?

	Never	Almost never (a few times a year or less)	Rarely (once a month or less)	Sometimes (a few times a month)	Often (once a week)	Very often (a few times a week)	Always (every day)
A. Enthusiastic							
B. Energetic							
C. Content							
D. At ease							
E. Anxious							
F. Angry							
G. Gloomy							
H. Discouraged							

Q13. How often do you experience fatigue when you are working?

- ☐ Never
- ☐ Almost never (a few times a year or less)
- ☐ Rarely (once a month or less)
- ☐ Sometimes (a few times a month)
- ☐ Often (once a week)
- ☐ Very often (a few times a week)
- ☐ Always (every day)

Q14. My work inspires me.

- ☐ Never
- ☐ Almost never (a few times a year or less)
- ☐ Rarely (once a month or less)
- ☐ Sometimes (a few times a month)
- ☐ Often (once a week)
- ☐ Very often (a few times a week)
- ☐ Always (every day)

Q15. I am immersed in my work.

- ☐ Never
- ☐ Almost never (a few times a year or less)
- ☐ Rarely (once a month or less)
- ☐ Sometimes (a few times a month)
- ☐ Often (once a week)
- ☐ Very often (a few times a week)
- ☐ Always (every day)

Q16. When I get up in the morning, I feel like going to work.

- ☐ Never
- ☐ Almost never (a few times a year or less)
- ☐ Rarely (once a month or less)
- ☐ Sometimes (a few times a month)
- ☐ Often (once a week)
- ☐ Very often (a few times a week)
- ☐ Always (every day)

The questions in this section ask how you feel about your organization and about benefits and health programs available at work. If you have more than one job, please answer questions as they apply to your *main* job.

Q17. At my organization, I am treated with respect.

- ☐ Strongly disagree
- ☐ Somewhat disagree
- ☐ Somewhat agree
- ☐ Strongly agree
- ☐ Does not apply

Q18. My organization values my contributions.

- ☐ Strongly disagree
- ☐ Somewhat disagree
- ☐ Somewhat agree
- ☐ Strongly agree
- ☐ Does not apply

Q19. My organization cares about my general satisfaction at work.

- ☐ Strongly disagree
- ☐ Somewhat disagree
- ☐ Somewhat agree
- ☐ Strongly agree
- ☐ Does not apply

Q20. My organization is willing to extend resources in order to help me perform my job to the best of my ability.

- ☐ Strongly disagree
- ☐ Somewhat disagree
- ☐ Somewhat agree
- ☐ Strongly agree
- ☐ Does not apply

Q21. I receive recognition for a job well done.

- Strongly disagree
- Somewhat disagree
- Somewhat agree
- Strongly agree

Q22. I trust the management at my organization.

- Strongly disagree
- Somewhat disagree
- Somewhat agree
- Strongly agree
- Does not apply

Q23. My organization is committed to employee health and well-being.

- Strongly disagree
- Somewhat disagree
- Somewhat agree
- Strongly agree
- Does not apply

Q24. My organization encourages me and provides opportunities to engage in healthy behaviors, such as being physically active, eating a healthy diet, living tobacco free, and managing my stress.

- Strongly disagree
- Somewhat disagree
- Somewhat agree
- Strongly agree
- Does not apply

Q25. Are the following benefits offered by your employer?

	Yes	No	Don't know	Does not apply
A. Health insurance				
B. Assistance with education/tuition				
C. Retirement (employer contributions to retirement savings)				
D. Paid maternity leave				
E. Paid paternity leave				
F. Paid sick leave				
G. Other paid caregiving leave (for example, to care for sick family members)				
H. Paid disability leave				
I. Paid vacation days				
J. Other paid leave (for example, bereavement, emergency, jury duty)				
K. Ability to take unpaid leave				
L. Transit options (such as help with transportation to and from work)				
M. On-site medical care				
N. Employee assistance programs (such as programs that help workers with personal or work-related problems)				

Q26. Are the following health and wellness programs or services available to you at the place where you work?

	Yes	No	Don't know	Does not apply
A. Health education and promotion programs (wellness programs)				
B. On-site fitness centers or gym membership discounts (includes a gym and/or space for group classes)				
C. Common spaces or activity hubs (areas for group activities, such as socializing, exercise classes, etc.)				
D. Smoking cessation programs				
E. Alcohol and substance programs				
F. Stress management programs				
G. Access to healthy lunch and snack options				

Q27. How often do the demands of your job interfere with your personal life?

- ☐ Never
- ☐ Almost never (a few times a year or less)
- ☐ Rarely (once a month or less)
- ☐ Sometimes (a few times a month)
- ☐ Often (once a week)
- ☐ Very often (a few times a week)
- ☐ Always (every day)

Q28. How often do the demands of your personal life interfere with your work on the job?

- ☐ Never
- ☐ Almost never (a few times a year or less)
- ☐ Rarely (once a month or less)
- ☐ Sometimes (a few times a month)
- ☐ Often (once a week)
- ☐ Very often (a few times a week)
- ☐ Always (every day)

Q29. I have the freedom to vary my work schedule.

- ☐ Strongly disagree
- ☐ Somewhat disagree
- ☐ Somewhat agree
- ☐ Strongly agree

Q30. I have the freedom to work wherever is best for me—either at home or at my organization.

- ☐ Strongly disagree
- ☐ Somewhat disagree
- ☐ Somewhat agree
- ☐ Strongly agree
- ☐ Does not apply

The questions in this section ask about physical characteristics of your work environment and safety conditions where you work. If you have more than one job, please answer questions as they apply to your *main* job.

Q31. Overall, how safe do you think your workplace is?

- ☐ Very unsafe
- ☐ Somewhat unsafe
- ☐ Somewhat safe
- ☐ Very safe

Q32. Please indicate how much you agree or disagree with each of the following statements about safety practices at your workplace.

	Strongly disagree	Somewhat disagree	Somewhat agree	Strongly agree	Does not apply
A. Management reacts quickly to solve the problem when told about safety hazards.					
B. Management insists on thorough and regular safety audits and inspections.					
C. Management provides all the equipment needed to do the job safely.					
D. Management invests a lot of time and money in safety training for workers.					
E. Management listens carefully to workers' ideas about improving safety.					
F. Management gives safety personnel the power they need to do their job.					

Q33. On my present job, this is how I feel about the following topics:

	Not at all satisfied	Not too satisfied	Somewhat satisfied	Very satisfied
A. The environmental conditions (heating, lighting, ventilation, etc.)				
B. The physical surroundings (for example, building infrastructure, work area layout, design)				
C. The pleasantness of the work environment				

Q33D. The accommodations for disabilities and/or special needs (wheelchair ramps, lactation rooms, etc.)

- ☐ Not at all satisfied
- ☐ Not too satisfied
- ☐ Somewhat satisfied
- ☐ Very satisfied
- ☐ Does not apply

Q34. I feel discriminated against in my job because of my age.

- ☐ Strongly disagree
- ☐ Somewhat disagree
- ☐ Somewhat agree
- ☐ Strongly agree

Q35. I feel discriminated against in my job because of my race or ethnic origin.

- ☐ Strongly disagree
- ☐ Somewhat disagree
- ☐ Somewhat agree
- ☐ Strongly agree

Q36. I feel discriminated against in my job because of my gender.

- ☐ Strongly disagree
- ☐ Somewhat disagree
- ☐ Somewhat agree
- ☐ Strongly agree

Q37. In the past 12 months, were you sexually harassed by anyone while you were on the job?

- ☐ Yes
- ☐ No

Q38. In the past 12 months, were you exposed to physical violence while you were on the job?

- ☐ Yes
- ☐ No

Q39. In the past 12 months, were you bullied, threatened, or harassed in any other way by anyone while you were on the job?

- ☐ Yes
- ☐ No

Q40. In the past 12 months, have you been in a situation where any of your superiors or coworkers put you down or were condescending to you, made demeaning remarks about you, or addressed you in unprofessional terms?

- ☐ Yes
- ☐ No
- ☐ Does not apply

The questions in this section ask about your physical and mental health and health-related behaviors.

Q41. Would you say that in general, your health is poor, fair, good, very good, or excellent?

- ☐ Poor
- ☐ Fair
- ☐ Good
- ☐ Very good
- ☐ Excellent

Q42. Now, thinking about your physical health, which includes physical illness and injury, during the past 30 days, for how many days was your physical health not good?

Enter number of days (0–30) [ ]

Q43. Have you ever had any of the following?

	Never	In the past	Have currently
A. Arthritis			
B. Other musculoskeletal disorders (for example, back pain, neck pain, other pain)			
C. Asthma			
D. Lung disease, other than asthma (for example, chronic obstructive pulmonary disease [COPD], chronic bronchitis, emphysema)			
E. Cancer			
F. Depression			
G. Diabetes			
H. Heart disease			
I. High blood pressure			

Q44. Have you ever had chronic insomnia?

- ☐ Never
- ☐ In the past
- ☐ Have currently

Q45. Now, thinking about your mental health, which includes stress, depression, anxiety, and problems with emotions, during the past 30 days, for how many days was your mental health not good?

Enter number of days (0–30) [ ]

Q46. How often do you experience stress with regard to the following topics?

	Never	Almost never (a few times a year or less)	Rarely (once a month or less)	Sometimes (a few times a month)	Often (once a week)	Very often (a few times a week)	Always (every day)
A. Your health							
B. Your finances							
C. Your family or social relationships							
D. Your work							

Q47. Over the last 2 weeks, how often have you been bothered by feeling down, depressed, or hopeless?

- ☐ Not at all
- ☐ Several days
- ☐ More than half the days
- ☐ Nearly every day

Q48. Over the last 2 weeks, how often have you been bothered by little interest or pleasure in doing things?

- ☐ Not at all
- ☐ Several days
- ☐ More than half the days
- ☐ Nearly every day

Q49. Over the last 2 weeks, how often have you been bothered by feeling nervous, anxious, or on edge?

- ☐ Not at all
- ☐ Several days
- ☐ More than half the days
- ☐ Nearly every day

Q50. Over the last 2 weeks, how often have you been bothered by not being able to stop or control worrying?

- ☐ Not at all
- ☐ Several days
- ☐ More than half the days
- ☐ Nearly every day

Q51. In a typical week, how many days do you get at least 20 minutes of *high intensity* physical activity? (High intensity activity lasts at least 10 minutes and increases your heart rate, makes you sweat, and may make you feel out of breath; examples are running, fast cycling, and strenuous, continuous lifting of heavy objects.)

Enter number of days (0–7) ________

Q52. In a typical week, how many days do you get at least 30 minutes of *moderate intensity* physical activity? (Moderate intensity activity lasts at least 10 minutes and requires more effort than is needed for typical everyday tasks; examples are brisk walking, gardening, and continuous lifting of light objects.)

Enter number of days (0–7) ________

Q53. Do you use any of the following tobacco products?

	Never	Not any more	Some days	Daily
A. Cigarettes				
B. Cigars				
C. Pipes				
D. Smokeless tobacco				
E. Electronic cigarettes				

Q54. How many drinks of alcoholic beverages do you have in a typical week? (One drink = one beer, glass of wine, shot of liquor, or mixed drink.)

Enter number of drinks

Q55. During the past year, how often have you had more than four drinks if you are a male, or more than three drinks if you are a female, on any single day? (One drink = one beer, glass of wine, shot of liquor, or mixed drink.)

- ☐ Never
- ☐ Once (1 day)
- ☐ A few times (2 or 3 days)
- ☐ Often (more than 3 days)

Q56. Think of the foods that are a part of your normal diet. How many servings of fruits and vegetables do you eat in a normal day?

(One serving is any of the following: 1 cup raw leafy greens [about the size of a small fist]; 1⁄2 cup of other vegetables [cooked or raw]; 1 medium piece of fruit [about the size of a baseball]; 1/2 cup chopped, cooked, or canned fruit; or 3⁄4 cup vegetable or fruit juice.)

- ☐ Less than 1 serving
- ☐ 1 serving
- ☐ 2 servings
- ☐ 3 servings
- ☐ 4 servings
- ☐ 5 or more servings

Q57. How many hours of sleep do you usually get at night? If you are a shift worker, how many hours of sleep do you get a day?

- ☐ 6 or fewer hours
- ☐ 7 hours
- ☐ 8 hours
- ☐ 9 or more hours

Q58. In the past 7 days, how often have you felt sleepy while at work?

- ☐ Never
- ☐ Rarely
- ☐ Sometimes
- ☐ Usually
- ☐ Always

Q59. Because of a physical, mental, or emotional condition, do you have serious difficulty concentrating, remembering, or making decisions?

- ☐ Not at all
- ☐ Slightly
- ☐ Moderately
- ☐ Extremely
- ☐ Does not apply/do not have condition

Q60. Are you limited in the kind or amount of work you can do because of a physical, mental, or emotional problem?

- ☐ Not at all
- ☐ Slightly
- ☐ Moderately
- ☐ Extremely
- ☐ Does not apply/do not have problem

Q61. In the past month…

	Never	Almost never (one time a month)	Rarely (once a week or less)	Sometimes (a few times a week)	Often (once a day)	Very often (a few times a day)	Always (every hour)
A. How often did you not concentrate enough on your work?							
B. How often did you find yourself not working as carefully as you should?							
C. How often did you not work at times when you were supposed to be working?							
D. How often did you get less done than other workers?							

Q62. During the past 12 months, did you experience any work-related injuries?

- ☐ Yes
- ☐ No

Q63. If you experienced any work-related injuries in the past 12 months, did any of them require any first aid or medical treatment, change in job activities, or lost time from work?

- ☐ Yes
- ☐ No
- ☐ Does not apply/was not injured in the past 12 months

The questions in this section ask about your experiences, feelings, and activities outside of work.

Q64. In general, how satisfied are you with your life?

- ☐ Not at all satisfied
- ☐ Not too satisfied
- ☐ Somewhat satisfied
- ☐ Very satisfied

Q65. How worried are you right now about not being able to maintain the standard of living you enjoy?

- ☐ Not worried at all
- ☐ Not too worried
- ☐ Moderately worried
- ☐ Very worried

Q66. How worried are you right now about not having enough income to pay your normal monthly bills?

- ☐ Not worried at all
- ☐ Not too worried
- ☐ Moderately worried
- ☐ Very worried

Q67. How often do you get the social and emotional support you need from friends, family, or others outside of work?

- ☐ Never
- ☐ Rarely
- ☐ Sometimes
- ☐ Always

Q68. In general, how often do you take part in any of the following activities outside of work?

	Never	Almost never (A few times a year or less)	Rarely (Once a month or less)	Sometimes (A few times a month)	Often (Once a week)	Very often (A few times a week)	Always (Every day)	Does not apply
A. Voluntary or charitable activities								
B. Domestic caregiving activities (for example, children, elderly or disabled relatives/ friends, but not in a volunteer or charity setting)								
C. Home maintenance tasks (for example, cooking, cleaning, repairs)								
D. Socializing with friends, family, others								
E. Taking training or education courses								
F. Sporting, cultural, or leisure activities								
G. Relaxation or planned solitary activities								

You have completed the NIOSH WellBQ. Thank you for your time!

Optional Items

The questions in this section ask about your current working arrangements, occupation, and the industrial sector in which you are working. If you have more than one job, please answer questions as they apply to your *main* job.

E1. How would you describe your work arrangement in your job?

- ☐ I am an independent contractor, an independent consultant, or a freelance worker.
- ☐ I am on call and work only when called to work.
- ☐ I am paid by a temporary agency.
- ☐ I work for a contractor who provides workers and services to others under contract.
- ☐ I am a regular, permanent employee.

E2. Is your job full-time or part-time?

- ☐ Full-time
- ☐ Part-time

E3. How long have you worked in your job?

- ☐ Less than 1 year
- ☐ 1–5 years
- ☐ 6–10 years
- ☐ 10–20 years
- ☐ More than 20 years

E4. Select the occupation that best describes the kind of work you do in your job.

- ☐ Architecture and Engineering
- ☐ Arts, Design, Entertainment, Sports, and Media
- ☐ Building and Grounds Cleaning and Maintenance
- ☐ Business and Financial Operations
- ☐ Computer and Mathematical
- ☐ Community and Social Service
- ☐ Construction and Extraction
- ☐ Education Instruction and Library
- ☐ Farming, Fishing, and Forestry
- ☐ Food Preparation and Serving Related
- ☐ Healthcare Practitioners and Technical
- ☐ Healthcare Support
- ☐ Installation, Maintenance, and Repair
- ☐ Legal
- ☐ Life, Physical, and Social Science
- ☐ Management
- ☐ Material Moving
- ☐ Military Specific
- ☐ Office and Administrative Support
- ☐ Personal Care and Service
- ☐ Production
- ☐ Protective Service
- ☐ Sales and Related
- ☐ Transportation
- ☐ Other (Please specify): ____________________________

E5. Select the kind of industry or business you work in for your job.

- ☐ Arts, Entertainment, and Recreation
- ☐ Accommodation and Food Services
- ☐ Administrative and Support and Waste Management
- ☐ Agriculture, Forestry, Fishing, and Hunting
- ☐ Construction
- ☐ Educational Services
- ☐ Finance and Insurance
- ☐ Health Care and Social Assistance
- ☐ Information
- ☐ Management of Companies and Enterprises
- ☐ Manufacturing
- ☐ Military
- ☐ Mining, Quarrying, and Oil and Gas Extraction
- ☐ Other Services, Except Public Administration
- ☐ Public Administration
- ☐ Professional, Scientific, and Technical Services
- ☐ Real Estate and Rental and Leasing
- ☐ Retail Trade
- ☐ Transportation and Warehousing
- ☐ Utilities
- ☐ Wholesale Trade
- ☐ Other (Please specify): ____________________________

The questions in this section ask for basic information about yourself.

D1. What is your age?

- ☐ 18–29
- ☐ 30–44
- ☐ 45–64
- ☐ 65 and older

D2. What is the highest level of school you have completed or the highest degree you have received?

- ☐ Less than high school
- ☐ High school/GED
- ☐ Some college
- ☐ Bachelor's degree or higher

D3. Do you consider yourself to be Hispanic or Latino?

- ☐ Yes
- ☐ No
- ☐ Refused
- ☐ Don't know

D4. What race or races do you consider yourself to be? Please select one or more of these categories.

- ☐ White
- ☐ Black/African American
- ☐ American Indian
- ☐ Alaska Native
- ☐ Native Hawaiian
- ☐ Other Pacific Islander
- ☐ Asian
- ☐ Some other race
- ☐ Refused
- ☐ Don't know

D5. Are you male or female?

- ☐ Male
- ☐ Female
- ☐ Refused
- ☐ Don't know

D6. Do you think of yourself as gay/lesbian or gay; straight, that is, not gay/lesbian; bisexual; something else; or you don't know the answer?

- ☐ Gay/lesbian
- ☐ Straight, that is, not gay/lesbian
- ☐ Bisexual
- ☐ Something else
- ☐ I don't know the answer
- ☐ Refused
- ☐ Don't know

D7. What was your entire household income last year, before taxes?

- ☐ <$20,000
- ☐ $20,000 to $34,999
- ☐ $35,000 to $49,999
- ☐ $50,000 to $74,999
- ☐ $75,000 to $99,999
- ☐ $100,000 to $149,999
- ☐ $150,000 to $199,999
- ☐ $200,000 or more

D8. Are you the head of your household?

- ☐ Yes
- ☐ No

D9. What is your current marital status?

- ☐ Married or living with partner
- ☐ Widowed
- ☐ Divorced
- ☐ Separated
- ☐ Never married

D10. How many dependents currently live in your household? Please enter the total number in each age category.

A. Total number of household members age 0 to 5 ☐

B. Total number of household members age 6 to 12 ☐

C. Total number of household members age 13 to 17 ☐

D. Total number of household members age 18 or older ☐

NIOSH WellBQ Item Source Table

NIOSH WellBQ Item	Source
**Section 1: Work Evaluation and Experience**	
**Job Satisfaction**	
Q1. Overall, I am _____ with my job.<br>☐ Not at all satisfied<br>☐ Not too satisfied<br>☐ Somewhat satisfied<br>☐ Very satisfied	Centers for Disease Control and Prevention [2010] Quality of Worklife Questionnaire, http://www.cdc.gov/niosh/topics/stress/qwlquest.html.<br><br>Quinn RP, Staines GL [1979]. The 1977 Quality of Employment Survey: descriptive statistics, with comparison data from the 1969-70 and the 1972-1973 surveys. Ann Arbor, MI: The University of Michigan.
**Wage Satisfaction**	
Q2. I am ____ with my wages.<br>☐ Not at all satisfied<br>☐ Not too satisfied<br>☐ Somewhat satisfied<br>☐ Very satisfied	Macdonald S, MacIntyre P [1997]. The generic job satisfaction scale: scale development and its correlates. Employee Assistance Quarterly *13*(2):1-16.
**Benefits Satisfaction**	
Q3. I am ____ with the benefits provided by my employer.<br>☐ Not at all satisfied<br>☐ Not too satisfied<br>☐ Somewhat satisfied<br>☐ Very satisfied<br>☐ Does not apply	Centers for Disease Control and Prevention [2010]. Quality of Worklife Questionnaire, http://www.cdc.gov/niosh/topics/stress/qwlquest.html.<br><br>Quinn RP, Staines GL [1979]. The 1977 Quality of Employment Survey: descriptive statistics, with comparison data from the 1969-70 and the 1972-1973 surveys. Ann Arbor, MI: The University of Michigan.
**Advancement Satisfaction**	
Q4. I am ____ with my chances for advancement on the job.<br>☐ Not at all satisfied<br>☐ Not too satisfied<br>☐ Somewhat satisfied<br>☐ Very satisfied	Vocational Psychology Research (VPR), University of Minnesota [1977]. Minnesota Satisfaction Questionnaire Long Form 1977, http://vpr.psych.umn.edu/

NIOSH WellBQ Item	Source
**Supervisor Support**	
Q5. I can count on my supervisor for support when I need it.<br>☐ Strongly disagree<br>☐ Somewhat disagree<br>☐ Somewhat agree<br>☐ Strongly agree<br>☐ Does not apply	Centers for Disease Control and Prevention [2010]. Quality of Worklife Questionnaire, http://www.cdc.gov/niosh/topics/stress/qwlquest.html.<br><br>Quinn RP, Staines GL [1979]. The 1977 Quality of Employment Survey: descriptive statistics, with comparison data from the 1969-70 and the 1972 -1973 surveys. Ann Arbor, MI: The University of Michigan.<br><br>Caplan RD, Cobb S, French JRP Jr, Van Harrison R, Pinnneau SR Jr [1975]. Job demands and worker health: main effects and occupational differences. DHEW (NIOSH) Publication No. 75-160.
**Coworker Support**	
Q6. I can count on my coworkers for support when I need it.<br>☐ Strongly disagree<br>☐ Somewhat disagree<br>☐ Somewhat agree<br>☐ Strongly agree<br>☐ Does not apply	Centers for Disease Control and Prevention [2010]. Quality of Worklife Questionnaire, http://www.cdc.gov/niosh/topics/stress/qwlquest.html.<br><br>Quinn RP, Staines GL [1979]. The 1977 Quality of Employment Survey: descriptive statistics, with comparison data from the 1969-70 and the 1972-1973 surveys. Ann Arbor, MI: The University of Michigan.<br><br>Caplan RD, Cobb S, French JRP Jr, Van Harrison R, Pinnneau SR Jr [1975]. Job demands and worker health: main effects and occupational differences. DHEW (NIOSH) Publication No. 75-160.
**Job Security**	
Q7. I feel my job is secure.<br>☐ Strongly disagree<br>☐ Somewhat disagree<br>☐ Somewhat agree<br>☐ Strongly agree	Macdonald S, MacIntyre P [1997]. The generic job satisfaction scale: scale development and its correlates. Employee Assistance Quarterly *13*(2):1-16.

NIOSH WellBQ Item	Source
**Job Autonomy**	
Q8. I am given a lot of freedom to decide how to do my own work.<br>☐ Strongly disagree<br>☐ Somewhat disagree<br>☐ Somewhat agree<br>☐ Strongly agree	Centers for Disease Control and Prevention [2010]. Quality of Worklife Questionnaire, http://www.cdc.gov/niosh/topics/stress/qwlquest.html.<br><br>Quinn RP, Staines GL [1979]. The 1977 Quality of Employment Survey: descriptive statistics, with comparison data from the 1969-70 and the 1972-1973 surveys. Ann Arbor, MI: The University of Michigan.
**Time Paucity/Work Overload**	
Q9. I never seem to have enough time to get everything done on my job.<br>☐ Strongly disagree<br>☐ Somewhat disagree<br>☐ Somewhat agree<br>☐ Strongly agree	Centers for Disease Control and Prevention [2010]. Quality of Worklife Questionnaire, http://www.cdc.gov/niosh/topics/stress/qwlquest.html.<br><br>Quinn RP, Staines GL [1979]. The 1977 Quality of Employment Survey: descriptive statistics, with comparison data from the 1969-70 and the 1972-1973 surveys. Ann Arbor, MI: The University of Michigan.
**Meaningful Work**	
Q10. The work I do is meaningful to me.<br>☐ Strongly disagree<br>☐ Somewhat disagree<br>☐ Somewhat agree<br>☐ Strongly agree	Steger MF, Dik BJ, Duffy RD [2012]. Measuring meaningful work: the Work and Meaning Inventory (WAMI). J Career Assessment *20*(3):322-337.
Q11. The work I do serves a greater purpose.<br>☐ Strongly disagree<br>☐ Somewhat disagree<br>☐ Somewhat agree<br>☐ Strongly agree	Steger MF, Dik BJ, Duffy RD [2012]. Measuring meaningful work: the Work and Meaning Inventory (WAMI). J Career Assessment *20*(3):322-337.

NIOSH WellBQ Item	Source
**Work-related Positive and Work-related Negative Affect**	
Q12. [Grid] How often do you experience these feelings when you are working?<br>**Statements in row:**<br>A. Enthusiastic<br>B. Energetic<br>C. Content<br>D. At ease<br>E. Anxious<br>F. Angry<br>G. Gloomy<br>H. Discouraged<br>**Responses in column:**<br>☐ Never<br>☐ Almost never (a few times a year or less)<br>☐ Rarely (once a month or less)<br>☐ Sometimes (a few times a month)<br>☐ Often (once a week)<br>☐ Very often (a few times a week)<br>☐ Always (every day)	Van Katwyk PT, Fox S, Spector PE, Kelloway EK [2000]. Using the Job-related Affective Well-being Scale (JAWS) to investigate affective responses to work stressors. J Occup Health Psych *5*(2):219-230.
**Work-related Fatigue**	
Q13. How often do you experience fatigue when you are working?<br>☐ Never<br>☐ Almost never (a few times a year or less)<br>☐ Rarely (once a month or less)<br>☐ Sometimes (a few times a month)<br>☐ Often (once a week)<br>☐ Very often (a few times a week)<br>☐ Always (every day)	Van Katwyk PT, Fox S, Spector PE, Kelloway EK [2000]. Using the Job-related Affective Well-being Scale (JAWS) to investigate affective responses to work stressors. J Occup Health Psych *5*(2):219-230.
**Job Engagement**	
Q14. My work inspires me.<br>☐ Never<br>☐ Almost never (a few times a year or less)<br>☐ Rarely (once a month or less)<br>☐ Sometimes (a few times a month)<br>☐ Often (once a week)<br>☐ Very often (a few times a week)<br>☐ Always (every day)	Schaufeli WB, Bakker AB, Salanova M [2006]. The measurement of work engagement with a short questionnaire: a cross-national study. Educational and Psychological Measurement *66*(4):702-716.

NIOSH WellBQ Item	Source
Q15. I am immersed in my work.<br><br>☐ Never<br>☐ Almost never (a few times a year or less)<br>☐ Rarely (once a month or less)<br>☐ Sometimes (a few times a month)<br>☐ Often (once a week)<br>☐ Very often (a few times a week)<br>☐ Always (every day)	Schaufeli WB, Bakker AB, Salanova M [2006]. The measurement of work engagement with a short questionnaire: a cross-national study. Educational and Psychological Measurement *66*(4):702-716.
Q16. When I get up in the morning, I feel like going to work.<br><br>☐ Never<br>☐ Almost never (a few times a year or less)<br>☐ Rarely (once a month or less)<br>☐ Sometimes (a few times a month)<br>☐ Often (once a week)<br>☐ Very often (a few times a week)<br>☐ Always (every day)	Schaufeli WB, Bakker AB, Salanova M [2006]. The measurement of work engagement with a short questionnaire: a cross-national study. Educational and Psychological Measurement *66*(4):702-716.
**Section 2: Workplace Policies and Culture**	
**Supportive Work Culture**	
Q17. At my organization, I am treated with respect.<br><br>☐ Strongly disagree<br>☐ Somewhat disagree<br>☐ Somewhat agree<br>☐ Strongly agree<br>☐ Does not apply	Centers for Disease Control and Prevention [2010]. Quality of Worklife Questionnaire, http://www.cdc.gov/niosh/topics/stress/qwlquest.html.
Q18. My organization values my contributions.<br><br>☐ Strongly disagree<br>☐ Somewhat disagree<br>☐ Somewhat agree<br>☐ Strongly agree<br>☐ Does not apply	Eisenberger R, Huntington R, Hutchinson S, Sowa D [1986]. Perceived organizational support. J Appl Psychol *71*(3):500-507, http://classweb.uh.edu/eisenberger/perceived-organizational-support/.
Q19. My organization cares about my general satisfaction at work.<br><br>☐ Strongly disagree<br>☐ Somewhat disagree<br>☐ Somewhat agree<br>☐ Strongly agree<br>☐ Does not apply	Eisenberger R, Huntington R, Hutchinson S, Sowa D [1986]. Perceived organizational support. J Appl Psychol *71*(3):500-507, http://classweb.uh.edu/eisenberger/perceived-organizational-support/.

NIOSH WellBQ Item	Source
Q20. My organization is willing to extend resources in order to help me perform my job to the best of my ability.<br>☐ Strongly disagree<br>☐ Somewhat disagree<br>☐ Somewhat agree<br>☐ Strongly agree<br>☐ Does not apply	Eisenberger R, Huntington R, Hutchinson S, Sowa D [1986]. Perceived organizational support. J Appl Psychol *71*(3):500-507, http://classweb.uh.edu/eisenberger/perceived-organizational-support/.
Q21. I receive recognition for a job well done.<br>☐ Strongly disagree<br>☐ Somewhat disagree<br>☐ Somewhat agree<br>☐ Strongly agree	Macdonald S, MacIntyre P [1997]. The generic job satisfaction scale: scale development and its correlates. Employee Assistance Quarterly *13*(2):1-16.
**Management Trust**	
Q22. I trust the management at my organization.<br>☐ Strongly disagree<br>☐ Somewhat disagree<br>☐ Somewhat agree<br>☐ Strongly agree<br>☐ Does not apply	Centers for Disease Control and Prevention [2010]. Quality of Worklife Questionnaire, http://www.cdc.gov/niosh/topics/stress/qwlquest.html.
**Health Culture at Work**	
Q23. My organization is committed to employee health and well-being.<br>☐ Strongly disagree<br>☐ Somewhat disagree<br>☐ Somewhat agree<br>☐ Strongly agree<br>☐ Does not apply	Zweber ZM, Henning RA, Magley VJ [2016]. A practical scale for Multi-Faceted Organizational Health Climate Assessment. J Occup Health Psychol *21*(2):250-259.
Q24. My organization encourages me and provides opportunities to engage in healthy behaviors, such as being physically active, eating a healthy diet, living tobacco free, and managing my stress.<br>☐ Strongly disagree<br>☐ Somewhat disagree<br>☐ Somewhat agree<br>☐ Strongly agree<br>☐ Does not apply	Zweber ZM, Henning RA, Magley VJ [2016]. A practical scale for Multi-Faceted Organizational Health Climate Assessment. J Occup Health Psychol *21*(2):250-259.

NIOSH WellBQ Item	Source
**Availability of Job Benefits**	
Q25. [Grid] Are the following benefits offered by your employer?<br>**Statements in row:**<br>A. Health insurance<br>B. Assistance with education/tuition<br>C. Retirement (employer contributions to retirement savings)<br>D. Paid maternity leave<br>E. Paid paternity leave<br>F. Paid sick leave<br>G. Other paid caregiving leave (for example, to care for sick family members)<br>H. Paid disability leave<br>I. Paid vacation days<br>J. Other paid leave (for example, bereavement, emergency, jury duty)<br>K. Ability to take unpaid leave<br>L. Transit options (such as help with transportation to and from work)<br>M. On-site medical care<br>N. Employee assistance programs (such as programs that help workers with personal or work-related problems)<br>**Responses in column:**<br>☐ Yes<br>☐ No<br>☐ Don't know<br>☐ Does not apply	New item

NIOSH WellBQ Item	Source
**Availability of Health Programs at Work**	
Q26. [Grid] Are the following health and wellness programs or services available to you at the place where you work?<br>**Statements in row:**<br>☐ Health education and promotion programs (wellness programs)<br>☐ On-site fitness centers or gym membership discounts (includes a gym and/or space for group classes)<br>☐ Common spaces or activity hubs (areas for group activities, such as socializing, exercise classes, etc.)<br>☐ Smoking cessation programs<br>☐ Alcohol and substance programs<br>☐ Stress management programs<br>☐ Access to healthy lunch and snack options<br>**Responses in column:**<br>☐ Yes<br>☐ No<br>☐ Don't know<br>☐ Does not apply	New item
**Work to Non-work Conflict**	
Q27. How often do the demands of your job interfere with your personal life?<br>☐ Never<br>☐ Almost never (a few times a year or less)<br>☐ Rarely (once a month or less)<br>☐ Sometimes (a few times a month)<br>☐ Often (once a week)<br>☐ Very often (a few times a week)<br>☐ Always (every day)	Centers for Disease Control and Prevention [2010]. Quality of Worklife Questionnaire, http://www.cdc.gov/niosh/topics/stress/qwlquest.html.<br><br>Quinn RP, Staines GL [1979]. The 1977 Quality of Employment Survey: descriptive statistics, with comparison data from the 1969-70 and the 1972-1973 surveys. Ann Arbor, MI: The University of Michigan.
**Non-work to Work Conflict**	
Q28. How often do the demands of your personal life interfere with your work on the job?<br>☐ Never<br>☐ Almost never (a few times a year or less)<br>☐ Rarely (once a month or less)<br>☐ Sometimes (a few times a month)<br>☐ Often (once a week)<br>☐ Very often (a few times a week)<br>☐ Always (every day)	Centers for Disease Control and Prevention [2010]. Quality of Worklife Questionnaire, http://www.cdc.gov/niosh/topics/stress/qwlquest.html.<br><br>Quinn RP, Staines GL [1979]. The 1977 Quality of Employment Survey: descriptive statistics, with comparison data from the 1969-70 and the 1972-1973 surveys. Ann Arbor, MI: The University of Michigan.

NIOSH WellBQ Item	Source
**Workplace/Schedule Flexibility**	
Q29. I have the freedom to vary my work schedule.<br>☐ Strongly disagree<br>☐ Somewhat disagree<br>☐ Somewhat agree<br>☐ Strongly agree	Shockley KM, Allen TD [2007]. When flexibility helps: another look at the availability of flexible work arrangements and work–family conflict. J Vocational Behavior *71*;479-493.
Q30. I have the freedom to work wherever is best for me—either at home or at my organization.<br>☐ Strongly disagree<br>☐ Somewhat disagree<br>☐ Somewhat agree<br>☐ Strongly agree<br>☐ Does not apply	Shockley KM, Allen TD [2007]. When flexibility helps: another look at the availability of flexible work arrangements and work–family conflict. J Vocational Behavior *71*;479-493.
**Section 3: Workplace Physical Environment and Safety Climate**	
**Overall Workplace Safety**	
Q31. Overall, how safe do you think your workplace is?<br>☐ Very unsafe<br>☐ Somewhat unsafe<br>☐ Somewhat safe<br>☐ Very safe	Centers for Disease Control and Prevention [ND]. CDC NHWP Health and Safety Climate Survey (INPUTS) user manual. National Center for Chronic Disease Prevention and Health Promotion, https://www.cdc.gov/workplacehealthpromotion/tools-resources/pdfs/NHWP_INPUTS_Manual.pdf.

NIOSH WellBQ Item	Source
**Workplace Safety Climate**	
Q32. [Grid] Please indicate how much you agree or disagree with each of the following statements about safety practices at your workplace.<br><br>**Statements in row:**<br>A. Management reacts quickly to solve the problem when told about safety hazards.<br>B. Management insists on thorough and regular safety audits and inspections.<br>C. Management provides all the equipment needed to do the job safely.<br>D. Management invests a lot of time and money in safety training for workers.<br>E. Management listens carefully to workers' ideas about improving safety.<br>F. Management gives safety personnel the power they need to do their job.<br><br>**Responses in column:**<br>☐ Strongly disagree<br>☐ Somewhat disagree<br>☐ Somewhat agree<br>☐ Strongly agree<br>☐ Does not apply	Zohar D, Luria G [2005]. A multilevel model of safety climate: cross-level relationships between organization and group-level climates. J Applied Psychol *90*(4):616–628.
**Physical Work Environment Satisfaction**	
Q33A-33C. [Grid] On my present job, this is how I feel about the following topics:<br><br>**Statements in row:**<br>A. The environmental conditions (heating, lighting, ventilation, etc.)<br>B. The physical surroundings (for example, building infrastructure, work area layout, design)<br>C. The pleasantness of the work environment<br><br>**Statements in column:**<br>☐ Not at all satisfied<br>☐ Not too satisfied<br>☐ Somewhat satisfied<br>☐ Very satisfied	Vocational Psychology Research (VPR), University of Minnesota [1977]. Minnesota Satisfaction Questionnaire Long Form 1977, http://vpr.psych.umn.edu/

NIOSH WellBQ Item	Source
Q33D. The accommodations for disabilities and/or special needs (wheelchair ramps, lactation rooms, etc.)<br>☐ Not at all satisfied<br>☐ Not too satisfied<br>☐ Somewhat satisfied<br>☐ Very satisfied<br>☐ Does not apply	New item
**Discrimination**	
Q34. I feel discriminated against in my job because of my age.<br>☐ Strongly disagree<br>☐ Somewhat disagree<br>☐ Somewhat agree<br>☐ Strongly agree	Centers for Disease Control and Prevention [2010]. Quality of Worklife Questionnaire, http://www.cdc.gov/niosh/topics/stress/qwlquest.html.<br><br>Quinn RP, Staines GL [1979]. The 1977 Quality of Employment Survey: descriptive statistics, with comparison data from the 1969-70 and the 1972-1973 surveys. Ann Arbor, MI: The University of Michigan.
Q35. I feel discriminated against in my job because of my race or ethnic origin.<br>☐ Strongly disagree<br>☐ Somewhat disagree<br>☐ Somewhat agree<br>☐ Strongly agree	Centers for Disease Control and Prevention [2010]. Quality of Worklife Questionnaire, http://www.cdc.gov/niosh/topics/stress/qwlquest.html.<br><br>Quinn RP, Staines GL [1979]. The 1977 Quality of Employment Survey: descriptive statistics, with comparison data from the 1969-70 and the 1972-1973 surveys. Ann Arbor, MI: The University of Michigan.
Q36. I feel discriminated against in my job because of my gender.<br>☐ Strongly disagree<br>☐ Somewhat disagree<br>☐ Somewhat agree<br>☐ Strongly agree	Centers for Disease Control and Prevention [2010]. Quality of Worklife Questionnaire, http://www.cdc.gov/niosh/topics/stress/qwlquest.html.<br><br>Quinn RP, Staines GL [1979]. The 1977 Quality of Employment Survey: descriptive statistics, with comparison data from the 1969-70 and the 1972-1973 surveys. Ann Arbor, MI: The University of Michigan.

NIOSH WellBQ Item	Source
**Work-related Sexual Harassment**	
Q37. In the past 12 months, were you sexually harassed by anyone while you were on the job?<br>☐ Yes<br>☐ No	Centers for Disease Control and Prevention [2010]. Quality of Worklife Questionnaire, http://www.cdc.gov/niosh/topics/stress/qwlquest.html.
**Work-related Physical Violence**	
Q38. In the past 12 months, were you exposed to physical violence while you were on the job?<br>☐ Yes<br>☐ No	National Research Centre for the Working Environment (NRCWE) [2005)]. Copenhagen Psychosocial Questionnaire—COPSOQ II. https://nfa.dk/da/Vaerktoejer/Sporgeskemaer/Copenhagen-Psychosocial-Questionnaire-COPSOQ-II/Engelsk-udgave.
**Work-related Bullying**	
Q39. In the past 12 months, were you bullied, threatened, or harassed in any other way by anyone while you were on the job?<br>☐ Yes<br>☐ No	Centers for Disease Control and Prevention [2010]. Quality of Worklife Questionnaire, http://www.cdc.gov/niosh/topics/stress/qwlquest.html.
Q40. In the past 12 months, have you been in a situation where any of your superiors or coworkers put you down or were condescending to you, made demeaning remarks about you, or addressed you in unprofessional terms?<br>☐ Yes<br>☐ No<br>☐ Does not apply	Cortina LM, Magley VJ, Williams JH, Langhout RD [2001]. Incivility in the workplace: incidence and impact. J Occup Health Psychol *6*(1):64.
**Section 4: Health Status**	
**Overall Health**	
Q41. Would you say that in general, your health is poor, fair, good, very good, or excellent?<br>☐ Poor<br>☐ Fair<br>☐ Good<br>☐ Very good<br>☐ Excellent	Centers for Disease Control and Prevention [2014]. Behavioral Risk Factor Surveillance System Questionnaire, http://www.cdc.gov/brfss/questionnaires/pdf-ques/2014_brfss.pdf.

NIOSH WellBQ Item	Source
**Days Poor Physical Health**	
Q42. Now, thinking about your physical health, which includes physical illness and injury, during the past 30 days, for how many days was your physical health not good?<br><br>Enter number of days (0–30)	Centers for Disease Control and Prevention [2014]. Behavioral Risk Factor Surveillance System Questionnaire, http://www.cdc.gov/brfss/questionnaires/pdf-ques/2014_brfss.pdf.
**Chronic Health Conditions**	
Q43. [Grid] Have you ever had any of the following?<br><br>**Statements in row:**<br>A. Arthritis<br>B. Other musculoskeletal disorders (for example, back pain, neck pain, other pain)<br>C. Asthma<br>D. Lung disease, other than asthma (for example, chronic obstructive pulmonary disease [COPD], chronic bronchitis, emphysema)<br>E. Cancer<br>F. Depression<br>G. Diabetes<br>H. Heart disease<br>I. High blood pressure<br><br>**Responses in column:**<br>☐ Never<br>☐ In the past<br>☐ Have currently	Items 43A and 43C–H: Health Enhancement Research Organization and Population Health Alliance [2015]. Program measurement and evaluation guide: core metrics for employee health management, https://hero-health. org/wp content/uploads/2015/02/HERO-PHA-Metrics-Guide-FINAL.pdf.<br><br>Items 43B and 43I are new.
**Insomnia**	
Q44. Have you ever had chronic insomnia?<br>☐ Never<br>☐ In the past<br>☐ Have currently	Health Enhancement Research Organization and Population Health Alliance [2015]. Program measurement and evaluation guide: core metrics for employee health management, https://hero-health. org/wp-content/uploads/2015/02/HERO-PHA-Metrics-Guide-FINAL.pdf.
**Days of Poor Mental Health**	
Q45. Now, thinking about your mental health, which includes stress, depression, anxiety, and problems with emotions, during the past 30 days, for how many days was your mental health not good?<br><br>Enter number of days (0–30) ☐	Centers for Disease Control and Prevention [2014]. Behavioral Risk Factor Surveillance System Questionnaire, http://www.cdc.gov/brfss/questionnaires/pdf-ques/2014_brfss.pdf.

NIOSH WellBQ Item	Source
**Overall Stress**	
Q46. [Grid] How often do you experience stress with regard to the following topics?<br>**Statements in row:**<br>A. Your health<br>B. Your finances<br>C. Your family or social relationships<br>D. Your work<br>**Responses in column:**<br>☐ Never<br>☐ Almost never (a few times a year or less)<br>☐ Rarely (once a month or less)<br>☐ Sometimes (a few times a month)<br>☐ Often (once a week)<br>☐ Very often (a few times a week)<br>☐ Always (every day)	Health Enhancement Research Organization and Population Health Alliance [2015]. Program measurement and evaluation guide: core metrics for employee health management, https://hero-health.org/wp-content/uploads/2015/02/HERO-PHA-Metrics-Guide-FINAL.pdf.
**Poor Mental Health**	
Q47. Over the last 2 weeks, how often have you been bothered by feeling down, depressed, or hopeless?<br>☐ Not at all<br>☐ Several days<br>☐ More than half the days<br>☐ Nearly everyday	Kroenke K, Spitzer RL, Williams JBW, Löwe B [2009]. An ultra-brief screening scale for anxiety and depression: the PHQ-4. Psychosomatics *50*:613-621.
Q48. Over the last 2 weeks, how often have you been bothered by little interest or pleasure in doing things?<br>☐ Not at all<br>☐ Several days<br>☐ More than half the days<br>☐ Nearly everyday	Kroenke K, Spitzer RL, Williams JBW, Löwe B [2009]. An ultra-brief screening scale for anxiety and depression: the PHQ-4. Psychosomatics *50*:613-621.
Q49. Over the last 2 weeks, how often have you been bothered by feeling nervous, anxious, or on edge?<br>☐ Not at all<br>☐ Several days<br>☐ More than half the days<br>☐ Nearly everyday	Kroenke K, Spitzer RL, Williams JBW, Löwe B [2009]. An ultra-brief screening scale for anxiety and depression: the PHQ-4. Psychosomatics *50*:613-621.

NIOSH WellBQ Item	Source
Q50. Over the last 2 weeks, how often have you been bothered by not being able to stop or control worrying?<br>☐ Not at all<br>☐ Several days<br>☐ More than half the days<br>☐ Nearly everyday	Kroenke K, Spitzer RL, Williams JBW, Löwe B [2009]. An ultra-brief screening scale for anxiety and depression: the PHQ-4. Psychosomatics *50*:613-621.
**Physical Activity**	
Q51. In a typical week, how many days do you get at least 20 minutes of *high intensity* physical activity? (High intensity activity lasts at least 10 minutes and increases your heart rate, makes you sweat, and may make you feel out of breath; examples are running, fast cycling, and strenuous, continuous lifting of heavy objects.)<br>Enter number of days (0–7) ☐	Health Enhancement Research Organization and Population Health Alliance [2015]. Program measurement and evaluation guide: core metrics for employee health management, https://hero-health.org/wp-content/uploads/2015/02/HERO-PHA-Metrics-Guide-FINAL.pdf.
Q52. In a typical week, how many days do you get at least 30 minutes of *moderate intensity* physical activity? (Moderate intensity activity lasts at least 10 minutes and requires more effort than is needed for typical everyday tasks; examples are brisk walking, gardening, and continuous lifting of light objects.)<br>Enter number of days (0–7) ☐	Health Enhancement Research Organization and Population Health Alliance [2015]. Program measurement and evaluation guide: core metrics for employee health management, https://hero-health.org/wp-content/uploads/2015/02/HERO-PHA-Metrics-Guide-FINAL.pdf.
**Tobacco Use**	
Q53. [Grid] Do you use any of the following tobacco products?<br>**Statements in row:**<br>A. Cigarettes<br>B. Cigars<br>C. Pipes<br>D. Smokeless tobacco<br>E. Electronic cigarettes<br>**Responses in column:**<br>☐ Never<br>☐ Not any more<br>☐ Some days<br>☐ Daily	Health Enhancement Research Organization and Population Health Alliance [2015]. Program measurement and evaluation guide: core metrics for employee health management, https://hero-health.org/wp-content/uploads/2015/02/HERO-PHA-Metrics-Guide-FINAL.pdf.

NIOSH WellBQ Item	Source
**Alcohol Consumption**	
Q54. How many drinks of alcoholic beverages do you have in a typical week? (One drink = one beer, glass of wine, shot of liquor, or mixed drink.)<br><br>Enter number of drinks ☐	Health Enhancement Research Organization and Population Health Alliance [2015]. Program measurement and evaluation guide: core metrics for employee health management, https://hero-health.org/wp-content/uploads/2015/02/HERO-PHA-Metrics-Guide-FINAL.pdf.
**Risky Drinking**	
Q55. During the past year, how often have you had more than four drinks if you are a <u>male</u>, or more than three drinks if you are a <u>female</u>, on any single day? (One drink = one beer, glass of wine, shot of liquor, or mixed drink.)<br>☐ Never<br>☐ Once (1 day)<br>☐ A few times (2–3 days)<br>☐ Often (more than 3 days)	Health Enhancement Research Organization and Population Health Alliance [2015]. Program measurement and evaluation guide: core metrics for employee health management, https://hero-health.org/wp-content/uploads/2015/02/HERO-PHA-Metrics-Guide-FINAL.pdf.
**Healthy Diet**	
Q56. Think of the foods that are a part of your normal diet. How many servings of fruits and vegetables do you eat in a normal day?<br>(One serving is any of the following: 1 cup raw leafy greens [about the size of a small fist]; 1⁄2 cup of other vegetables [cooked or raw]; 1 medium piece of fruit [about the size of a baseball]; 1/2 cup chopped, cooked, or canned fruit; or 3⁄4 cup vegetable or fruit juice.)<br>☐ Less than 1 serving<br>☐ 1 serving<br>☐ 2 servings<br>☐ 3 servings<br>☐ 4 servings<br>☐ 5 or more servings	Health Enhancement Research Organization and Population Health Alliance [2015]. Program measurement and evaluation guide: core metrics for employee health management, https://hero-health.org/wp-content uploads/2015/02/HERO PHA-Metrics-Guide-FINAL.pdf.
**Sleep Hours**	
Q57. How many hours of sleep do you usually get at night? If you are a shift worker, how many hours of sleep do you get a day?<br>☐ 6 or fewer hours<br>☐ 7 hours<br>☐ 8 hours<br>☐ 9 or more hours	Health Enhancement Research Organization and Population Health Alliance [2015]. Program measurement and evaluation guide: core metrics for employee health management, https://hero-health.org/wp-content uploads/2015/02/HERO PHA-Metrics-Guide-FINAL.pdf.

NIOSH WellBQ Item	Source
**Sleepy at Work**	
Q58. In the past 7 days, how often have you felt sleepy while at work?<br>☐ Never<br>☐ Rarely<br>☐ Sometimes<br>☐ Usually<br>☐ Always	Health Enhancement Research Organization and Population Health Alliance [2015]. Program measurement and evaluation guide: core metrics for employee health management, https://hero-health.org/wp-content uploads/2015/02/HERO PHA-Metrics-Guide-FINAL.pdf.
**Cognitive Functioning Limitations**	
Q59. Because of a physical, mental, or emotional condition, do you have serious difficulty concentrating, remembering, or making decisions?<br>☐ Not at all<br>☐ Slightly<br>☐ Moderately<br>☐ Extremely<br>☐ Does not apply/do not have condition	Centers for Disease Control and Prevention [2014]. Behavioral Risk Factor Surveillance System Questionnaire, http://www.cdc.gov/brfss/questionnaires/pdf-ques/2014_brfss.pdf.
**Work Limitations**	
Q60. Are you limited in the kind or amount of work you can do because of a physical, mental, or emotional problem?<br>☐ Not at all<br>☐ Slightly<br>☐ Moderately<br>☐ Extremely<br>☐ Does not apply/do not have problem	Centers for Disease Control and Prevention [ND]. National Health and Nutrition Examination Survey. National Center for Health Statistics, https://www.cdc.gov/nchs/nhanes/.

NIOSH WellBQ Item	Source
**Productivity**	
Q61. [Grid] In the past month…<br>**Responses in rows:**<br>A. How often did you not concentrate enough on your work?<br>B. How often did you find yourself not working as carefully as you should?<br>C. How often did you not work at times when you were supposed to be working?<br>D. How often did you get less done than other workers?<br>**Responses in column:**<br>☐ Never<br>☐ Almost never (one time a month)<br>☐ Rarely (once a week or less)<br>☐ Sometimes (a few times a week)<br>☐ Often (once a day)<br>☐ Very often (a few times a day)<br>☐ Always (every hour)	The World Health Organization Health and Work Performance Questionnaire (HPQ) [2010], http://www.hcp.med.harvard.edu/hpq/info.php.
**Work-related Injury**	
Q62. During the past 12 months, did you experience any work-related injuries?<br>☐ Yes<br>☐ No	New item
**Injury Consequence**	
Q63. If you experienced any work-related injuries in the past 12 months, did any of them require any first aid or medical treatment, change in job activities, or lost time from work?<br>☐ No<br>☐ Yes<br>☐ Does not apply/was not injured in the past 12 months	New item
**Section 5: Home, Community, and Society**	
**Life Satisfaction**	
Q64. In general, how satisfied are you with your life?<br>☐ Not at all satisfied<br>☐ Not too satisfied<br>☐ Somewhat satisfied<br>☐ Very satisfied	Centers for Disease Control and Prevention [2014]. Behavioral Risk Factor Surveillance System Questionnaire, http://www.cdc.gov/brfss/questionnaires/pdf-ques/2014_brfss.pdf.

NIOSH WellBQ Item	Source
**Financial Insecurity**	
Q65. How worried are you right now about not being able to maintain the standard of living you enjoy?<br>☐ Not worried at all<br>☐ Not too worried<br>☐ Moderately worried<br>☐ Very worried	Centers for Disease Control and Prevention [ND]. National Health Interview Survey: NHIS Data, Questionnaires and Related Documentation, http://www.cdc.gov/nchs/nhis/data-questionnaires-documentation.htm.
Q66. How worried are you right now about not having enough income to pay your normal monthly bills?<br>☐ Not worried at all<br>☐ Not too worried<br>☐ Moderately worried<br>☐ Very worried	Centers for Disease Control and Prevention [ND]. National Health Interview Survey: NHIS Data, Questionnaires and Related Documentation, http://www.cdc.gov/nchs/nhis/data-questionnaires-documentation.htm.
**Support Outside of Work**	
Q67. How often do you get the social and emotional support you need from friends, family, or others outside of work?<br>☐ Never<br>☐ Rarely<br>☐ Sometimes<br>☐ Always	Centers for Disease Control and Prevention [2014]. Behavioral Risk Factor Surveillance System Questionnaire, http://www.cdc.gov/brfss/questionnaires/pdf-ques/2014_brfss.pdf.

NIOSH WellBQ Item	Source
**Activities Outside of Work**	
Q68. [Grid] In general, how often do you take part in any of the following activities outside of work?<br>**Statements in row:**<br>A. Voluntary or charitable activities<br>B. Domestic caregiving activities (for example, children, elderly or disabled relatives/friends, but not in a volunteer or charity setting)<br>C. Home maintenance tasks (for example, cooking, cleaning, repairs)<br>D. Socializing with friends, family, others<br>E. Taking training or education courses<br>F. Sporting, cultural, or leisure activities<br>G. Relaxation or planned solitary activities<br>**Responses in column:**<br>☐ Never<br>☐ Almost never (a few times a year or less)<br>☐ Rarely (Once a month or less)<br>☐ Sometimes (a few times a month)<br>☐ Often (once a week)<br>☐ Very often (a few times a week)<br>☐ Always (every day)<br>☐ Does not apply	Items 68A, 68B, 68E, and 68F:<br>European Foundation for the Improvement of Living and Working Conditions [2015]. 6th European Working Conditions Survey (EWCS): questionnaire, http://www.eurofound.europa.eu/sites/default/files/page/field_ef_documents/6th_ewcs_2015_final_source_master_questionnaire.pdf. (Permission is not needed to use items from the EWCS, as noted on the website.)<br><br>Items 68C, 68D, and 68G are new.

Optional Employment Item	Source
**Work Arrangement**	
E1. How would you describe your work arrangement in your job?<br>☐ I am an independent contractor, independent consultant, or a freelance worker.<br>☐ I am on call and work only when called to work.<br>☐ I am paid by a temporary agency.<br>☐ I work for a contractor who provides workers and services to others under contract.<br>☐ I am a regular, permanent employee.	Centers for Disease Control and Prevention [2010]. Quality of Worklife Questionnaire, http://www.cdc.gov/niosh/topics/stress/qwlquest.html.
**Work Status**	
E2. Is your job full-time or part-time?<br>☐ Full-time<br>☐ Part-time	New item
**Job Tenure**	
E3. How long have you worked in your job?<br>☐ Less than 1 year<br>☐ 1–5 years<br>☐ 6–10 years<br>☐ 10–20 years<br>☐ More than 20 years	New item
**Occupation**	
E4. Select the occupation that best describes the kind of work you do in your job.<br>[For full response list, please refer to the instrument]	U.S. Census Bureau [2010]. 2010 Census occupation codes with crosswalk. Download from the list at https://www.census.gov/topics/employment/ industry-occupation/guidance/code-lists.html.
**Industry or Business**	
E5. Select the kind of industry or business you work in for your job.<br>[For full response list, please refer to the instrument]	U.S. Census Bureau [2012]. Census 2012 detailed industry code list. Download from the list at https://www.census.gov/topics/employment/ industry-occupation/guidance/code-lists.html.

Optional Demographic Item	Source
**Age**	
D1. What is your age?<br>☐ 18–29<br>☐ 30–44<br>☐ 45–64<br>☐ 65 and older	New item
**Education**	
D2. What is the highest level of school you have completed or the highest degree you have received?<br>☐ Less than high school<br>☐ High school/GED<br>☐ Some college<br>☐ Bachelor's degree or higher	New item
**Ethnicity**	
D3. Do you consider yourself to be Hispanic or Latino?<br>☐ Yes<br>☐ No<br>☐ Refused<br>☐ Don't know	Centers for Disease Control and Prevention [ND]. National Health Interview Survey: NHIS Data, Questionnaires and Related Documentation, http://www.cdc.gov/nchs/nhis/data-questionnaires-documentation.htm.
**Race**	
D4. What race or races do you consider yourself to be? Please select one or more of these categories.<br>☐ White<br>☐ Black/African American<br>☐ American Indian<br>☐ Alaska Native<br>☐ Native Hawaiian<br>☐ Other Pacific Islander<br>☐ Asian<br>☐ Some other race<br>☐ Refused<br>☐ Don't know	Centers for Disease Control and Prevention [ND]. National Health Interview Survey: NHIS Data, Questionnaires and Related Documentation, http://www.cdc.gov/nchs/nhis/data-questionnaires-documentation.htm.

Optional Demographic Item	Source
**Sex**	
D5. Are you male or female?<br>☐ Male<br>☐ Female<br>☐ Refused<br>☐ Don't know	Centers for Disease Control and Prevention [ND]. National Health Interview Survey: NHIS Data, Questionnaires and Related Documentation, http://www.cdc.gov/nchs/nhis/data-questionnaires-documentation.htm.
**Sexual Orientation**	
D6. Do you think of yourself as gay/lesbian or gay; straight, that is, not gay/lesbian; bisexual; something else; or you don't know the answer?<br>☐ Gay/lesbian<br>☐ Straight, that is, not gay/lesbian<br>☐ Bisexual<br>☐ Something else<br>☐ I don't know the answer<br>☐ Refused<br>☐ Don't know	Centers for Disease Control and Prevention [ND]. National Health Interview Survey: NHIS Data, Questionnaires and Related Documentation, http://www.cdc.gov/nchs/nhis/data-questionnaires-documentation.htm.
**Household Income**	
D7. What was your entire household income last year, before taxes?<br>☐ <$20,000<br>☐ $20,000 to $34,999<br>☐ $35,000 to $49,999<br>☐ $50,000 to $74,999<br>☐ $75,000 to $99,999<br>☐ $100,000 to $149,999<br>☐ $150,000 to $199,999<br>☐ $200,000 or more	New item
**Head of Household**	
D8. Are you the head of your household?<br>☐ Yes<br>☐ No	New item

Optional Demographic Item	Source
**Marital Status**	
D9. What is your current marital status?<br>☐ Married or living with partner<br>☐ Widowed<br>☐ Divorced<br>☐ Separated<br>☐ Never married	New item
**Dependents**	
D10. How many dependents currently live in your household? Please enter the total number in each age category.<br><br>A. Total number of household members age 0 to 5 ______<br>B. Total number of household members age 6 to 12 ______<br>C. Total number of household members age 13 to 17 ______<br>D. Total number of household members age 18 or older ______	New item

Promoting productive workplaces through safety and health research

DHHS (NIOSH) Publication No. 2021-110 (Revised 05/2024)
DOI: https://doi.org/10.26616/NIOSHPUB2021110revised052024
